# Differences in Alpha Diversity of Gut Microbiota in Neurological Diseases

**DOI:** 10.3389/fnins.2022.879318

**Published:** 2022-06-28

**Authors:** Zhuoxin Li, Jie Zhou, Hao Liang, Li Ye, Liuyan Lan, Fang Lu, Qing Wang, Ting Lei, Xiping Yang, Ping Cui, Jiegang Huang

**Affiliations:** ^1^Guangxi Key Laboratory of AIDS Prevention and Treatment, Guangxi Universities Key Laboratory of Prevention and Control of Highly Prevalent Disease, Nanning, China; ^2^School of Public Health, Guangxi Medical University, Nanning, China; ^3^Life Science Institute, Guangxi Medical University, Nanning, China; ^4^Geriatrics Digestion Department of Internal Medicine, The First Affiliated Hospital of Guangxi Medical University, Nanning, China

**Keywords:** neurological diseases, alpha diversity, gut microbiota, 16S rRNA, gut microbiota-brain

## Abstract

**Background:**

Neurological diseases are difficult to diagnose in time, and there is currently a lack of effective predictive methods. Previous studies have indicated that a variety of neurological diseases cause changes in the gut microbiota. Alpha diversity is a major indicator to describe the diversity of the gut microbiota. At present, the relationship between neurological diseases and the alpha diversity of the gut microbiota remains unclear.

**Methods:**

We performed a systematic literature search of Pubmed and Bioproject databases up to January 2021. Six indices were used to measure alpha diversity, including community richness (observed species, Chao1 and ACE), community diversity (Shannon, Simpson), and phylogenetic diversity (PD). Random-effects meta-analyses on the standardized mean difference (SMD) were carried out on the alpha diversity indices. Subgroup analyses were performed to explore the sources of interstudy heterogeneity. Meta-analysis was performed on articles by matching the age, sex, and body mass index (BMI) of the disease group with the control group. Meanwhile, subgroup analysis was performed to control the variability of the sequencing region, platform, geographical region, instrument, and diseases. The area under the curve (AUC) value of the receiver operating characteristic (ROC) curve was calculated to assess the prediction effectiveness of the microbial alpha diversity indices.

**Results:**

We conducted a meta-analysis of 24 published studies on 16S rRNA gene amplified sequencing of the gut microbiota and neurological diseases from the Pubmed and Bioproject database (patients, *n* = 1,469; controls, *n* = 1,289). The pooled estimate demonstrated that there was no significant difference in the alpha diversity between patients and controls (*P* < 0.05). Alpha diversity decreased only in Parkinson's disease patients, while it increased in anorexia nervosa patients compared to controls. After adjusting for age, sex, BMI, and geographical region, none of the alpha diversity was associated with neurological diseases. In terms of Illumina HiSeq 2000 and the V3-V5 sequencing region, the results showed that alpha diversity increased significantly in comparison with the controls, while decreased in Illumina HiSeq 2500. ROC curves suggested that alpha diversity could be used as a biomarker to predict the AD (Simpson, AUC= 0.769, *P* = 0.0001), MS (observed species, AUC= 0.737, *P* = 0.001), schizophrenia (Chao1, AUC = 0.739, *P* = 0.002).

**Conclusions:**

Our review summarized the relationship between alpha diversity of the gut microbiota and neurological diseases. The alpha diversity of gut microbiota could be a promising predictor for AD, schizophrenia, and MS, but not for all neurological diseases.

## Introduction

Neurological diseases are neurological or psychiatric complications resulting from structural or functional abnormalities of the brain or spinal cord caused by various factors, which can be divided into two categories: neurodegeneration and neuropsychiatric diseases. Neurodegeneration includes Alzheimer's disease (AD), Parkinson's disease, Huntington's disease (HD), multiple sclerosis (MS), amyotrophic lateral sclerosis (ALS), motor neuron disease (MND), hepatic encephalopathy, neuromyelitis optica, chronic fatigue syndrome (CFS), Guillain-Barre syndrome, epilepsy, and cerebral ischemia. The other includes depressive disorder, post-traumatic stress disorder (PTSD), attention deficit hyperactivity disorder (ADHD), autism spectrum disorder (ASD), anorexia nervosa, schizophrenia, and anxiety. Neurological diseases are a type of disease with complex etiology, easy recurrence, and difficult timely diagnosis. Their prevalence in developing countries is at least twice that in developed countries (Kundap et al., 2017). The significantly growing incidence implies that the health of a large number of people is undermined and the economic, societal, and mental burden becomes substantial (Zhang et al., 2020). Regarding the serious hazards of neurological diseases, early clinical detection and disease prediction are important. However, neurological diseases are difficult to diagnose in time, and there is currently a lack of effective prediction methods.

A healthy gut microbiota participates in the body's growth, development, and aging process, and has the functions of improving human metabolism, regulating immunity, anti-inflammatory, anti-oxidant, anti-aging, and so on (Borre et al., 2014; Zhu et al., 2017). New evidence suggests that the gut microbiota may play an important role in the pathophysiology of various complex diseases (Jiang et al., 2015; Vogt and Kerby, 2017; Qian et al., 2018; Xu et al., 2020; Zou et al., 2020), including schizophrenia, depression, ASD, AD, and Parkinson's Disease, through the “gut microbiota-brain” axis (Morais et al., 2021). The gut microbiota-brain axis might have an important impact on the central nervous system through immunity, neuroendocrine, or vagus pathways. Nevertheless, there is still a lack of comprehensive and systematic understanding of the correlation between the pathophysiological mechanism of neurological diseases and the gut microbiota.

In a certain niche, the important features of a bacterial community include the number of species present, their numerical composition, and bacterial diversity (Mack et al., 2016). The Irish ELDERMET study showed that the greater the diversity of the microbiota, the better the health outcomes (Cryan et al., 2020). Alpha diversity is used to describe the microbial diversity of an ecological community (Willis, 2019). Generally, three indices were used to describe it. Community richness is a metric to estimate the total number of species, including the Chao 1, abundance-based coverage estimator (ACE) index, and observed species (the number of different operational taxonomic units, OTUs, per sample) (Finotello et al., 2018; Hagerty et al., 2020; Qian et al., 2020). Community diversity is a metric of relative community evenness, reflecting the richness and evenness of the species in the sample, including the Shannon index and Simpson index (Finotello et al., 2018; Hagerty et al., 2020; Qian et al., 2020). Another indicator is phylogenetic diversity (PD) (Owen et al., 2019), which is the sum of the length of the interconnected phylogenetic branches of a group of species in their phylogenetic tree (Gumbs et al., 2020).

Alpha diversity, as the most common indicator for assessing gut microbiota health, is closely associated with the disease status. Biodiversity plays an important role in maintaining a balanced ecosystem by contributing to the stability of an ecosystem as well as its ecological function (Gong et al., 2016). In general, a high diversity provides the ecosystem with strong stability (Gong et al., 2016). Wang et al. (2021) demonstrated that alpha diversity and *Bacteroides* abundance had the potential of identifying platinum-resistant patients early. Nylund et al. found that the severity of eczema was inversely correlated with the microbiota diversity (Nylund et al., 2015). Additionally, the microbial diversity was found to increase with the improvement of symptoms in eczema (Nylund et al., 2015).

Numerous studies have demonstrated that diseases of the central nervous system cause changes in the gut microbiota, but there is still no consensus on alpha diversity. Some studies revealed an increase in alpha diversity in ASD, epilepsy, or transient ischemic patients (Yin et al., 2015; Coretti et al., 2018; Huang et al., 2019; Dan et al., 2020). While other studies found it decreased in AD, hepatic encephalopathy, MS, or Parkinson's disease patients (Zhang et al., 2013; Chen et al., 2016; Iebba et al., 2018; Liu et al., 2019; Weis et al., 2019; Choileáin et al., 2020). Moreover, some researchers suggested that no significant differences were observed in the alpha diversity of the gut microbiota in patients with MS, schizophrenia, Parkinson's disease, ASD, or AD compared with healthy controls (Jangi, 2016; Pulikkan et al., 2018; Gong et al., 2019; Li et al., 2019; Nguyen et al., 2019; Pietrucci et al., 2019; Pan et al., 2020).

Importantly, it has not yet been determined whether the diversity of the gut microbiota can be used as an indicator to diagnose neurological diseases or predict the occurrence and development of the diseases. Aims to clarify the relationship between the diversity of the gut microbiota and neurological diseases, we performed a meta-analysis of gut microbiota studies on AD, Parkinson's disease, HD, MS, et, al. We comprehensively analyzed the alpha diversity index of the gut microbiota among neurological disease patients and controls, including estimates of community richness (Chao1, ACE, and observed species), community diversity (Shannon, Simpson), and PD.

## Materials and Methods

### Search Strategy and Selection Criteria

We performed a systematic literature search in the Pubmed database (https://pubmed.ncbi.nlm.nih.gov) and Bioproject database (https://www.ncbi.nlm.nih.gov/bioproject), up to January 2021, by using the following retrieval type “Alzheimer's disease ^*^[Title/Abstract] AND (gut or intestinal) ^*^[Title/Abstract] AND (microbe or microbiota or flora or microorganism or microbiota) ^*^[Title/Abstract]”. In order to more comprehensively search the relevant literature, the disease names in the above search formula were replaced successively by “Parkinson's disease”, “Huntington's disease”, “multiple sclerosis”, “amyotrophic lateral sclerosis”, “hepatic encephalopathy”, “motor neuron disease”, “chronic fatigue syndrome”, “Guillain-Barre syndrome”, “epilepsy”, “cerebral ischemia”, “depressive disorder”, “posttraumatic stress disorder”, “attention deficit hyperactivity disorder”, “autism spectrum disorder”, “anorexia nervosa”, “schizophrenia”, “anxiety”, and “neuromyelitis optica”.

**Figure 1:**
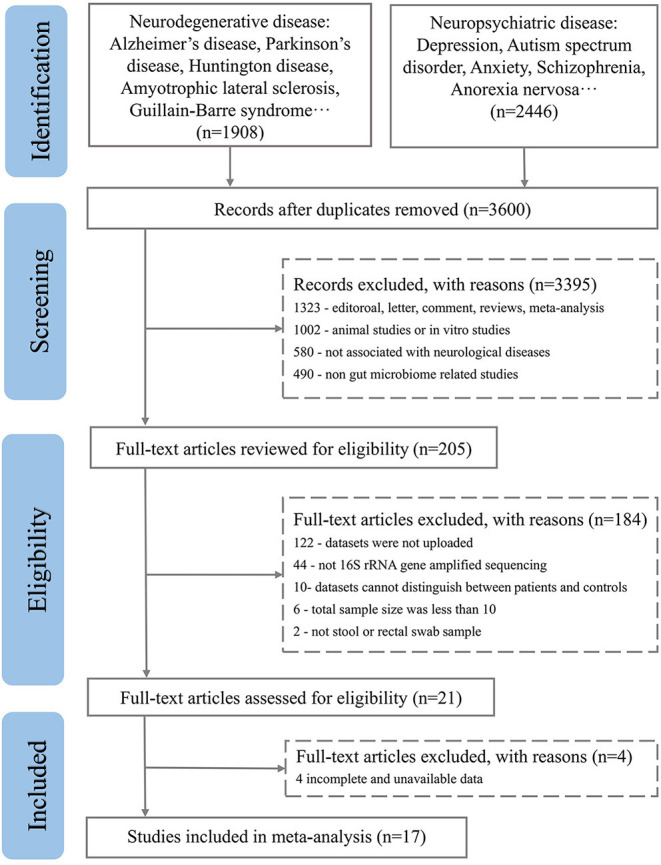
PRISMA flow diagram depicting the screening process from the Pubmed database for inclusion of studies.

**Figure 2:**
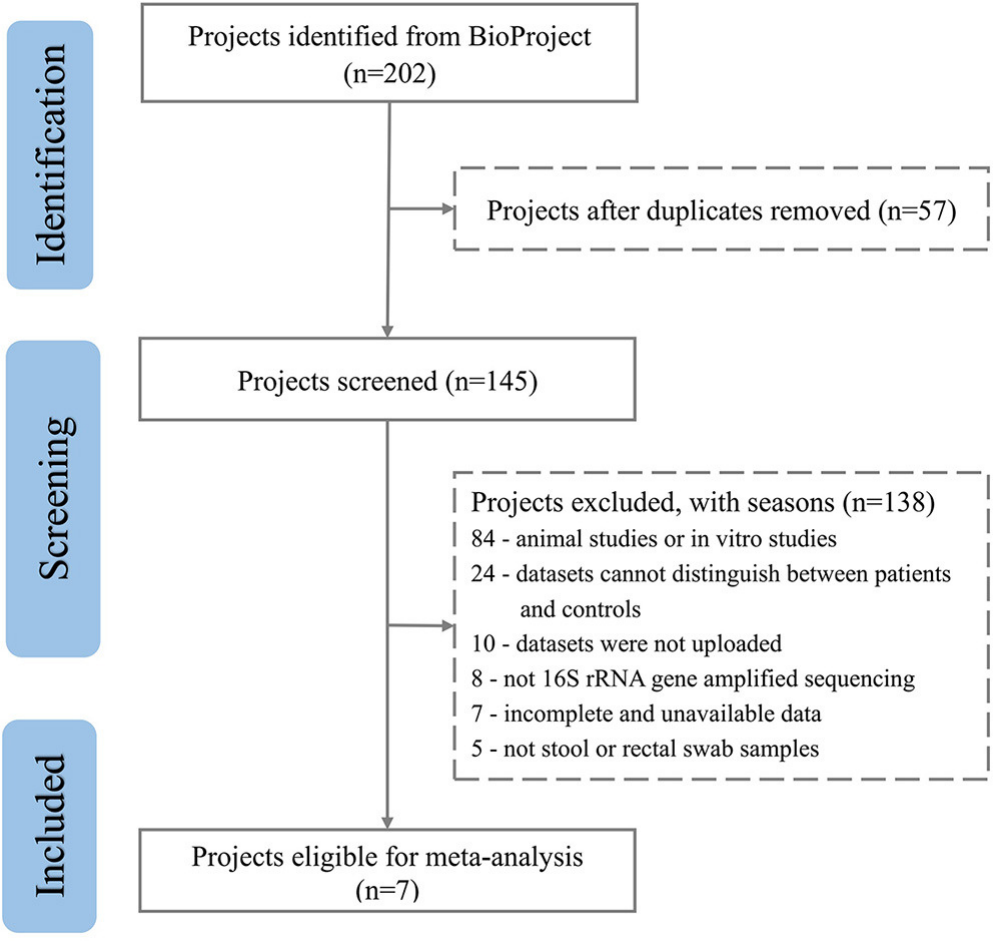
PRISMA flow diagram depicting the screening process from the Bioproject database for inclusion of studies.

After excluding comments and duplicate articles, the abstracts were filtered according to the inclusion and exclusion criteria. The inclusion criteria were: (1) Population study; (2) The source of the sample is a stool or rectal swab; (3) Using 16S rRNA gene amplified sequencing method; (4) The sample size cannot be less than 10 of each group; (5) Patient and control groups can be distinguished; (6) Availability of gut microbiota data. The exclusion criteria were: (1) Editorial, letter, comment, reviews, and meta-analysis; (2) Non gut microbiota-related studies and other diseases; (3) Animal studies or *in vitro* studies; (4) Unpublished data and studies that couldn't distinguish between patient and control groups. PRISMA flow diagram depicts the screening process from the Pubmed and Bioproject database for inclusion of studies (Figures 1, 2).

### Data Analysis

Fastq files for each study were downloaded from public databases. Microbiota bioinformatics was performed with QIIME 2 2021.2 (Bolyen et al., 2019). Raw sequence data were demultiplexed and quality filtered using the q2-demux plugin followed by denoising with DADA2 (Callahan et al., 2016), and then amplification sequence variants (ASVs) were generated. All amplicon sequence variants (ASVs) were aligned with mafft (Katoh et al., 2002) (*via* q2-alignment) and used to construct a phylogeny with fasttree2 (Price et al., 2010) (*via* q2-phylogeny). The amplicon sequence variants were annotated using the SilvA-138 99% OTU reference sequence pre-trained with the classify-Sklearn Naive Bayes classifier (McDonald et al., 2012) and the Q2 -feature-classifier (Bokulich et al., 2018) plug-in. Data filtering was performed to remove low-quality or uninformative features to improve downstream statistical analysis. Due to sequencing errors or contamination in the samples, we filtered the minimum count and prevalence (%) of the samples based on the characteristics of each dataset to obtain an analyzable dataset.

The generated feature table, phylogenetic tree, and species annotation files were imported into R Studio 4.0 software. The Vegan package was first used to normalize the sample sequence according to the minimum sequence number of samples so that the results, such as the alpha diversity of each sample, could be compared under the same sequence number. Then, the microeco package (Liu et al., 2021) was used to calculate the microbial alpha diversity index, including estimates of community richness (observed species, Chao1, and ACE), community diversity (Shannon, Simpson), and PD (Qian et al., 2020).

As primary outcomes of interest, we extracted the alpha diversity of the gut microbiota at the OTU level. Meta-analysis was performed on the included studies by using Review Manager 5.3 software. For continuous variables, mean and standard deviation (SD) were used as analytical statistics, and each effect size was represented by a 95% CI value. The heterogeneity of the included studies was tested by the *q-*value test and *I*^2^ test. Random-effects meta-analyses on the standardized mean difference (SMD) were executed for alpha diversity indices when each study had statistical heterogeneity (*Q-*value test of *P* < 0.05 or *I*^2^ test of >50%). Otherwise, the fixed-effect model was adopted. To explore sources of interstudy heterogeneity, the subgroup analysis controlled for age, sex, body mass index (BMI), sequencing region, geographical region, platform, instrument, and diseases. The area under the curve (AUC) value of the receiver operating characteristic (ROC) curve was calculated to assess the prediction effectiveness of the microbial alpha diversity indices. *P* < 0.05 was considered statistically significant.

## Results

### Characteristics of Selected Studies

A total of 3,600 records were retrieved from the PubMed database, according to the screening criteria, 21 articles (Zhang et al., 2013; Yin et al., 2015; Chen et al., 2016; Jangi, 2016; Mack et al., 2016; Coretti et al., 2018; Forbes et al., 2018; Iebba et al., 2018; Pulikkan et al., 2018; Gong et al., 2019; Huang et al., 2019; Li et al., 2019; Liu et al., 2019; Nguyen et al., 2019; Pietrucci et al., 2019; Weis et al., 2019; Choileáin et al., 2020; Dan et al., 2020; Pan et al., 2020; Safak et al., 2020; Cao et al., 2021) were included for analysis after eliminating the repeated, review, meta, and other types of articles. A total of 17 separate datasets were obtained from 17 articles (Zhang et al., 2013; Jangi, 2016; Mack et al., 2016; Coretti et al., 2018; Forbes et al., 2018; Pulikkan et al., 2018; Gong et al., 2019; Li et al., 2019; Liu et al., 2019; Nguyen et al., 2019; Pietrucci et al., 2019; Weis et al., 2019; Choileáin et al., 2020; Dan et al., 2020; Pan et al., 2020; Safak et al., 2020; Cao et al., 2021) (the remaining 4 articles (Yin et al., 2015; Chen et al., 2016; Iebba et al., 2018; Huang et al., 2019) were removed due to incomplete and unavailable data) (see Supplementary Table 1,2). At the same time, a total of 200 studies were retrieved from the BioProject by using the same retrieval type as the PubMed database. Finally, 7 datasets were included in the analysis (Table 1). Multiple case groups in the dataset were divided for subsequent meta-analysis, and 32 sub-datasets were finally obtained.

**Table 1:** Characteristics of studies included in Bioproject.

**Country, Year, Project description**	**BioProject accession**	**Study setting**	**Study population**	**Sample type**
China, 2020 Alterations of the fecal microbiota in Chinese patients with multiple sclerosis	PRJNA628832	Zhejiang University	34 MS	Fecal samples
China, 2019 In this study, four patients of cirrhosis accompanied with hepatic encephalopathy (CHE) were enrolled, with their healthy relatives as the controls, and paired comparison analysis was carried out. Fresh fecal samples were collected from participants, and subjected to Illumina MiSeq high throughput sequencing of 16S rRNA regions.	PRJNA534155	Institute of Disease Control and Prevention	4 hepatic encephalopathy; healthy controls (*n* = 4)	Fecal samples
USA, 2020 C. bolteae is elevated in NMOSD in India and shares sequence similarity with AQP4	PRJNA662563	Brigham and Women's Hospital	39 NMOSD patients; 36 matched controls	Fecal samples
China, 2020 gut microbiota in children with autism spectrum disorder accompanied with sleep disorder	PRJNA615774	Chongqing Medical University affiliated Children Hospital	ND	Fecal samples
South America Ecuador, 2019 Analysis of the intestinal and oropharyngeal microbiome, nutritional and immunological profile of children with Autism Spectrum Disorder (ASD) compared with children without	PRJEB27306	Universidad San Francisco de Quito	ND	Fecal samples
Korea, 2020 Gut microbiota of Human with mild anxiety/depression	PRJNA678145	Kyung Hee University	ND	Fecal samples
Japan 2020 Specific bacterial species relating to the development and progression of multiple sclerosis	PRJDB7752	Laboratory for Microbiome Sciences, Center for Integrative Medical Sciences, RIKEN	ND	Fecal samples

*ND, not described; NMOSD, neuromyelitis optica spectrum disorders; MS, multiple sclerosis*.

Neurological diseases, AD, epilepsy, hepatic encephalopathy, ASD, MS, Parkinson's disease, anorexia nervosa, optic neuromyelitis, and schizophrenia were included in the study. A total of 2,758 subjects (patient, *n* = 1469; control, *n* = 1289) samples were included in the meta-analysis. Twenty four studies compared patients with healthy controls, and 6 compared one or more subgroups with diverse clinical statuses. A total of 22 studies were conducted in Asia (China, Turkey, India, Korea, and Japan), 4 in Europe (Italy and Germany), 1 in South America, and 5 in North America (the United States of America and Ecuador). In addition, 22 studies used 16S rRNA gene amplified sequencing with the Illumina Miseq platform, 3 studies used Illumina Hiseq 2500, 2 studies used Illumina Hiseq 2000 sequences, and the remaining studies used Roche 454 pyrosequencing Illumina iSeq 100 and NextSeq 500.

### No Significant Change in the Diversity of Patients With Whole Neurological Diseases

Six indices were used to evaluate alpha diversity, including community richness (observed species, Chao1, ACE), community diversity (Shannon, Simpson), and PD. Before controlling for other confounders, the pooled estimate demonstrated that there is no significant difference between AD, epilepsy, hepatic encephalopathy, ASD, MS, Parkinson's disease, anorexia nervosa, optic neuromyelitis, and schizophrenia patients and controls, including observed species (SMD = −0.02; 95%CI, −0.16 to 0.12, *P* = 0.77, *I*^2^ = 64%), Chao1 (SMD = −0.02; 95%CI, −0.16 to 0.12, *P* = 0.79, *I*^2^ = 65%), and ACE (SMD = −0.07; 95%CI, −0.21 to 0.07, *P* = 0.34, *I*^2^ = 61%), Shannon (SMD = 0.00; 95%CI, −0.14 to 0.15, *P* = 0.98, *I*^2^ = 67%), Simpson (SMD = −0.00; 95%CI, −0.14 to 0.14, *P* = 0.99, *I*^2^ = 64%), PD (SMD = 0.02; 95%CI, −0.11 to 0.16, *P* = 0.74, *I*^2^ = 64%) (see Supplementary Figure 1).

### Community Diversity Altered in Parkinson's Disease and Anorexia Nervosa

**Figure 3:**
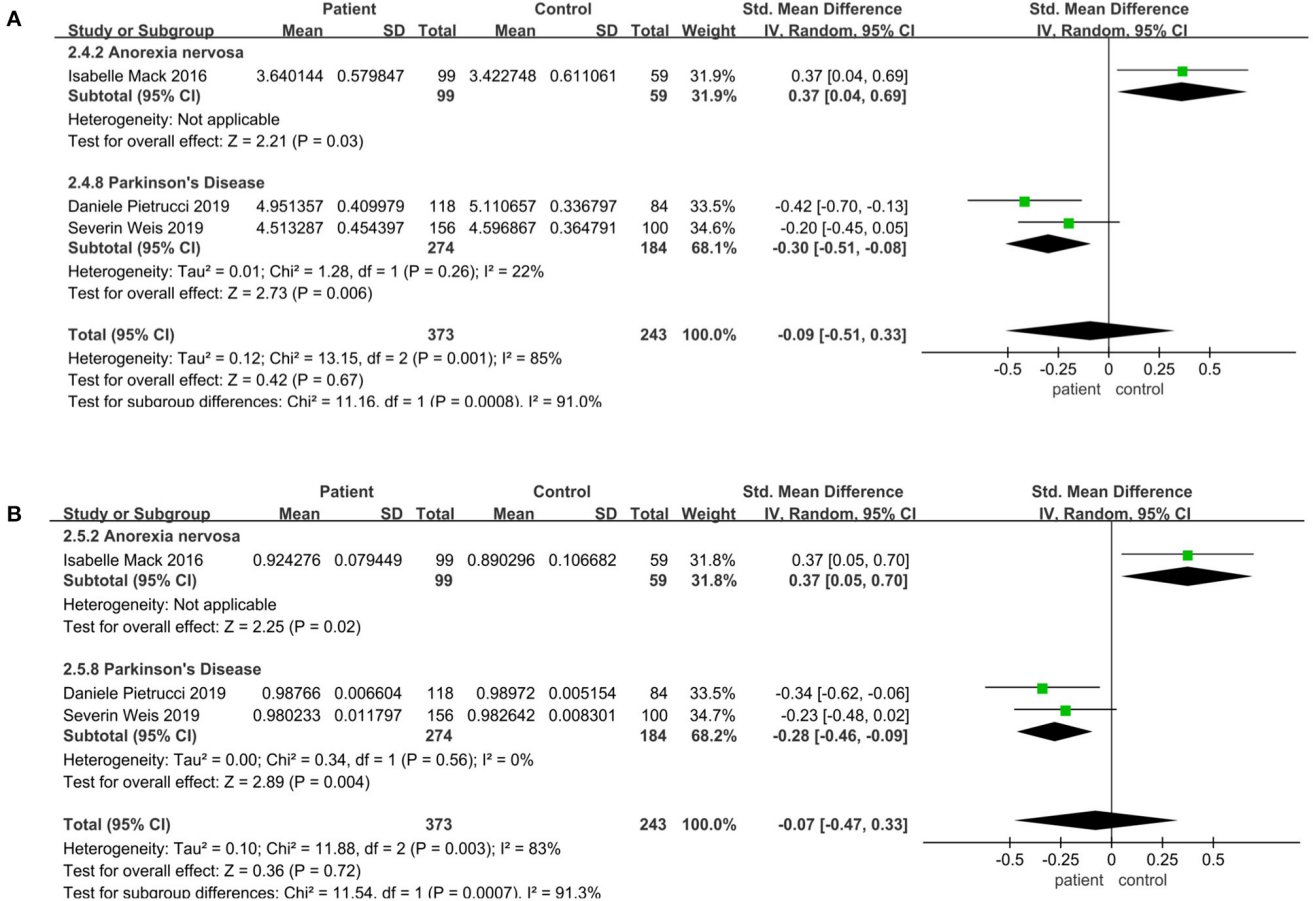
Community diversity meta-analyses for different diseases. Forest plot for the Shannon **(A)** and Simpson **(B)**. SMD, standardized mean difference; SD, standard deviation.

In terms of different diseases, there was a significant decrease in Parkinson's disease compared to the control, including Shannon (SMD = −0.30; 95%CI, −0.51 to −0.08, *P* = 0.0001, *I*^2^ = 22%) (Figure 3A), Simpson (SMD = −0.28; 95%CI, −0.46 to −0.09, *P* = 0.004, *I*^2^ = 0%) (Figure 3B). However, Shannon (SMD = 0.37; 95%CI, 0.04 to 0.69, *P* = 0.03) (Figure 3A) and Simpson (SMD = 0.37; 95%CI, 0.05 to 0.70, *P* = 0.03) (Figure 3B) increased significantly in anorexia nervosa. But the results demonstrated that there was no significant difference in the alpha diversity between patients and controls in other diseases (*P* > 0.05) (see Supplementary Figure 2).

### Community Richness Altered in the Illumine HiSeq Sequencing Instrument

Meta-analysis was performed on articles matching age, sex, and BMI between the patient and the control group. And subgroup analyses were performed to explore the sources of interstudy heterogeneity. However, the pooled estimate demonstrated that there is no significant difference between these two groups. When the analysis was limited to sequencing platform and geographical region, the result still showed no significant difference.

**Figure 4:**
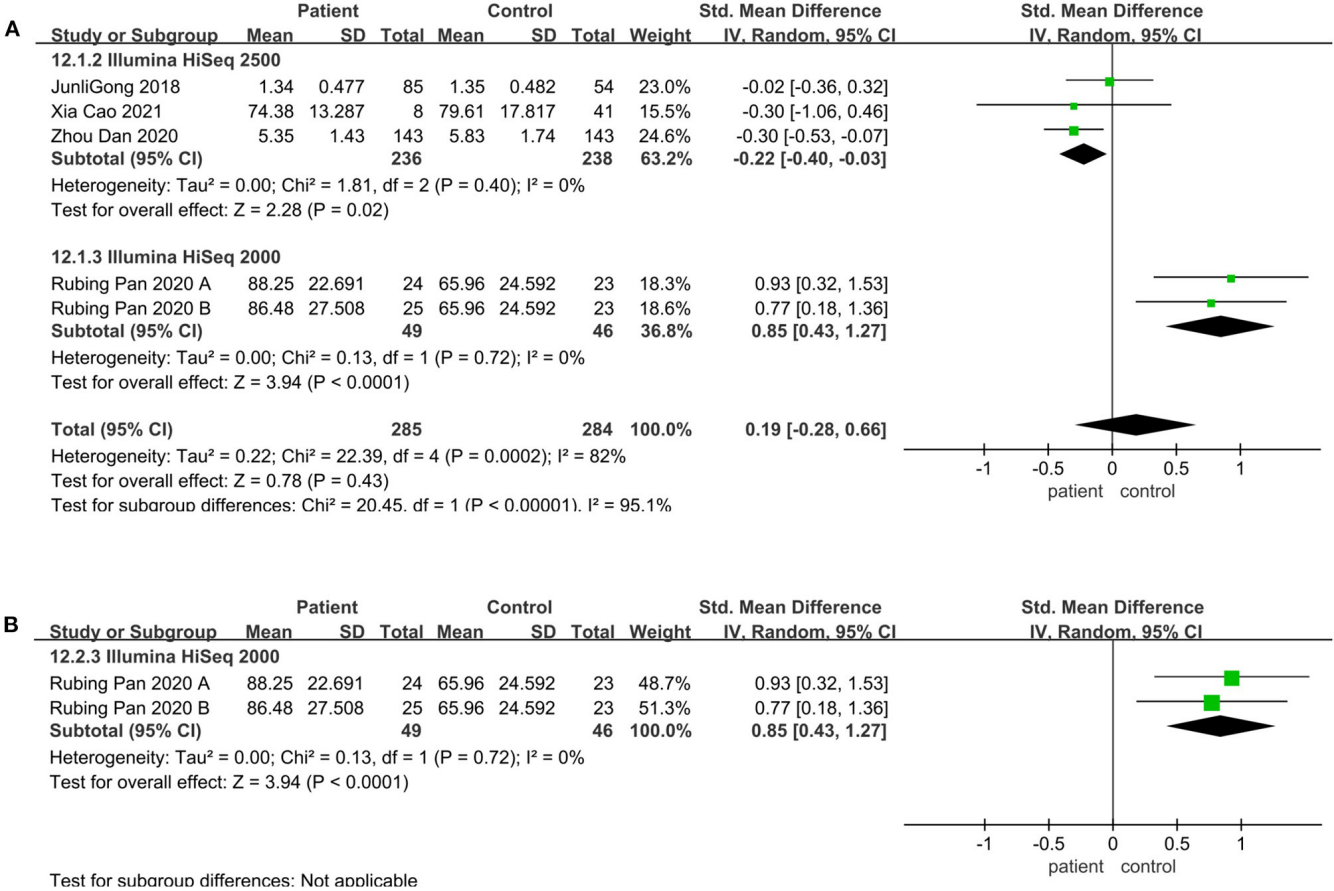
Richness meta-analyses for the sequencing instrument. Forest plot for the observed species **(A)** and Chao1 **(B)**. SMD, standardized mean difference; SD, standard deviation.

**Figure 5:**
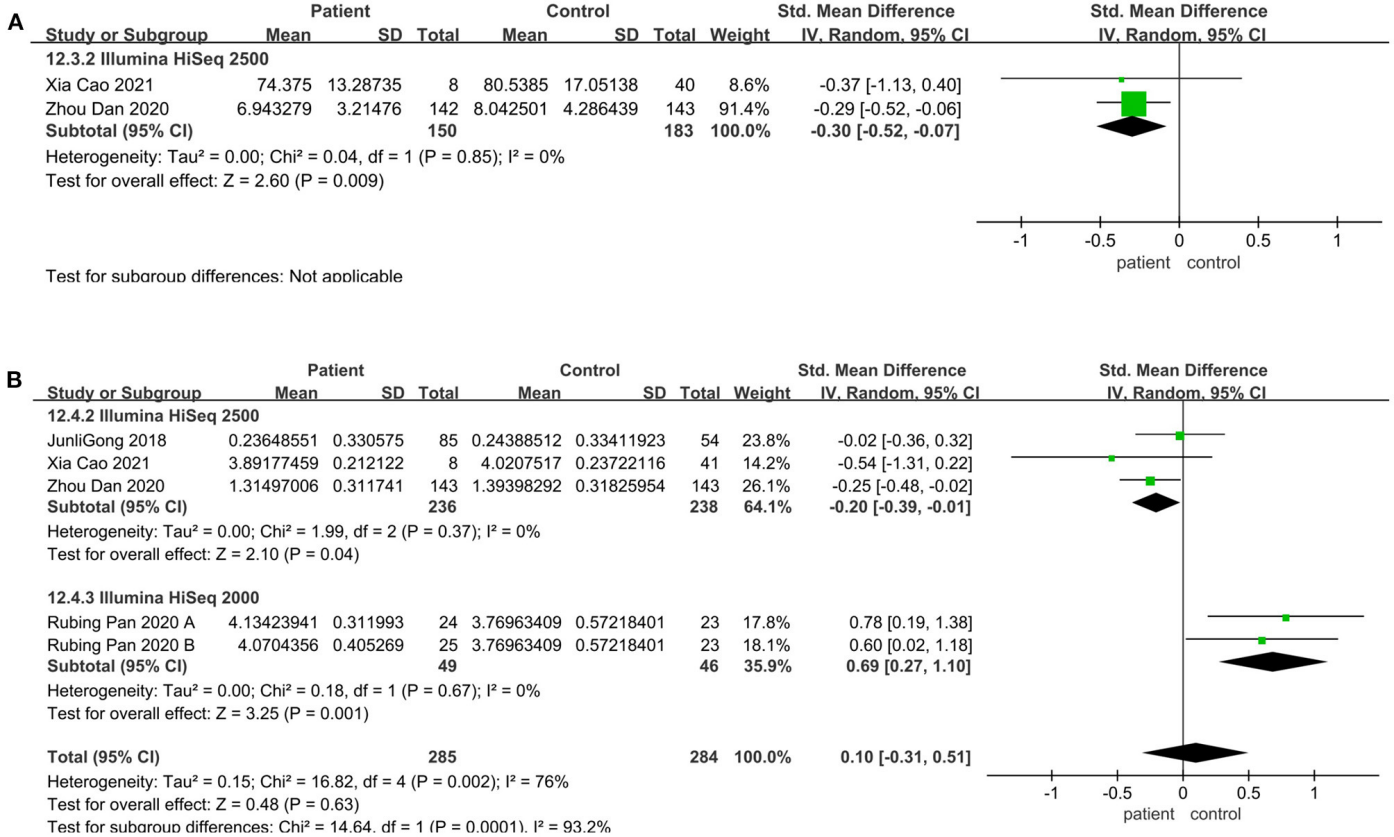
Richness meta-analyses for the sequencing instrument. Forest plot for the ACE **(A)** and Shannon **(B)**. SMD, standardized mean difference; SD, standard deviation.

**Figure 6:**
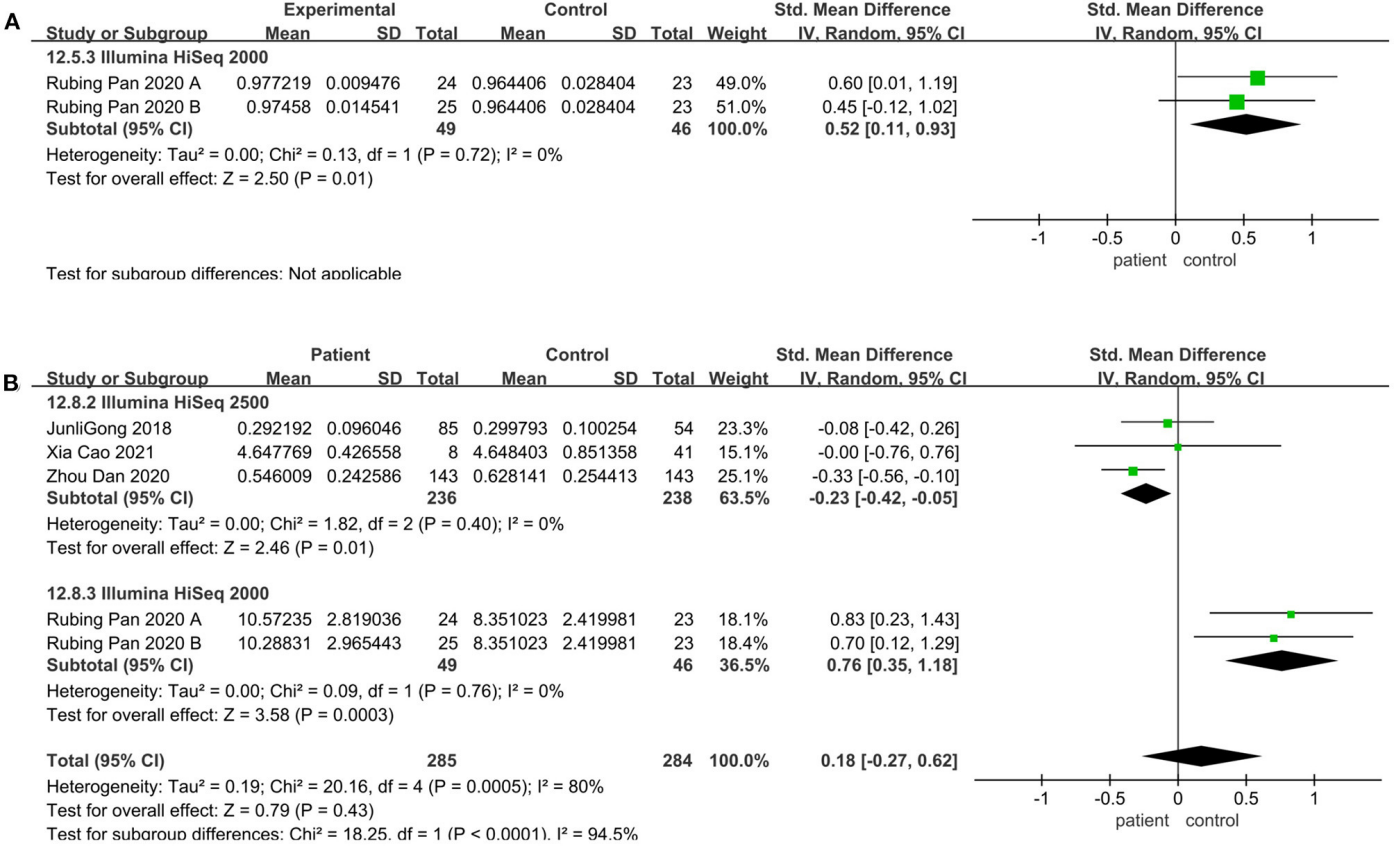
Evenness meta-analyses for the sequencing instrument. Forest plot for the Simpson **(A)**, and PD **(B)**. SMD, standardized mean difference; SD, standard deviation.

Regarding the sequencing instrument, 32 studies provided observed species, Chao1, Shannon, and Simpson indices. The pooled estimate showed that no significant difference were found, including Shannon (SMD = 0.00; 95%CI, −0.14 to 0.15, *P* = 0.98, *I*^2^ = 67%) (see Supplementary Figure 3) and Simpson (SMD = −0.01; 95%CI, −0.16 to 0.13, *P* = 0.84, *I*^2^ = 66%) (see Supplementary Figure 4). Within Illumine HiSeq 2500, there was a significant decrease in the patient group compared to the control group (observed species SMD = −0.22; 95%CI, −0.40 to −0.03; *P* = 0.02, *I*^2^ = 0%; ACE SMD = −0.30; 95%CI, −0.52 to −0.07; *P* = 0.009, *I*^2^ = 0%; Shannon SMD = −0.20; 95%CI, −0.39 to −0.01; *P* = 0.04, *I*^2^ = 0%; PD SMD = −0.23; 95%CI, −0.42 to −0.05; *P* = 0.01, *I*^2^ = 0%) (Figures 4A, 5A,B, 6B). But in Illumine HiSeq 2000, the alpha diversity in the patient group was significantly increased in the observed species (SMD = 0.85; 95%CI, 0.43 to 1.27, *P*= 0.0001, *I*^2^ = 0%) (Figure 4A), Chao1 (SMD = 0.85; 95%CI, 0.43 to 1.27, *P*=0.0001, *I*^2^ = 0%) (Figure 4B), Shannon (SMD = 0.69; 95%CI, 0.27 to 1.10, *P* = 0.001, *I*^2^ = 0%) (Figure 5B), Simpson (SMD = 0.52; 95%CI, 0.11 to 0.93, *P* = 0.01, *I*^2^ = 0%) (Figure 6A), and PD (SMD = 0.76; 95%CI, 0.35 to 1.18, *P* = 0.0003, *I*^2^ = 0%) (Figure 6B).

### Alpha Diversity Altered in the V3-V5 Sequencing Region

**Figure 7:**
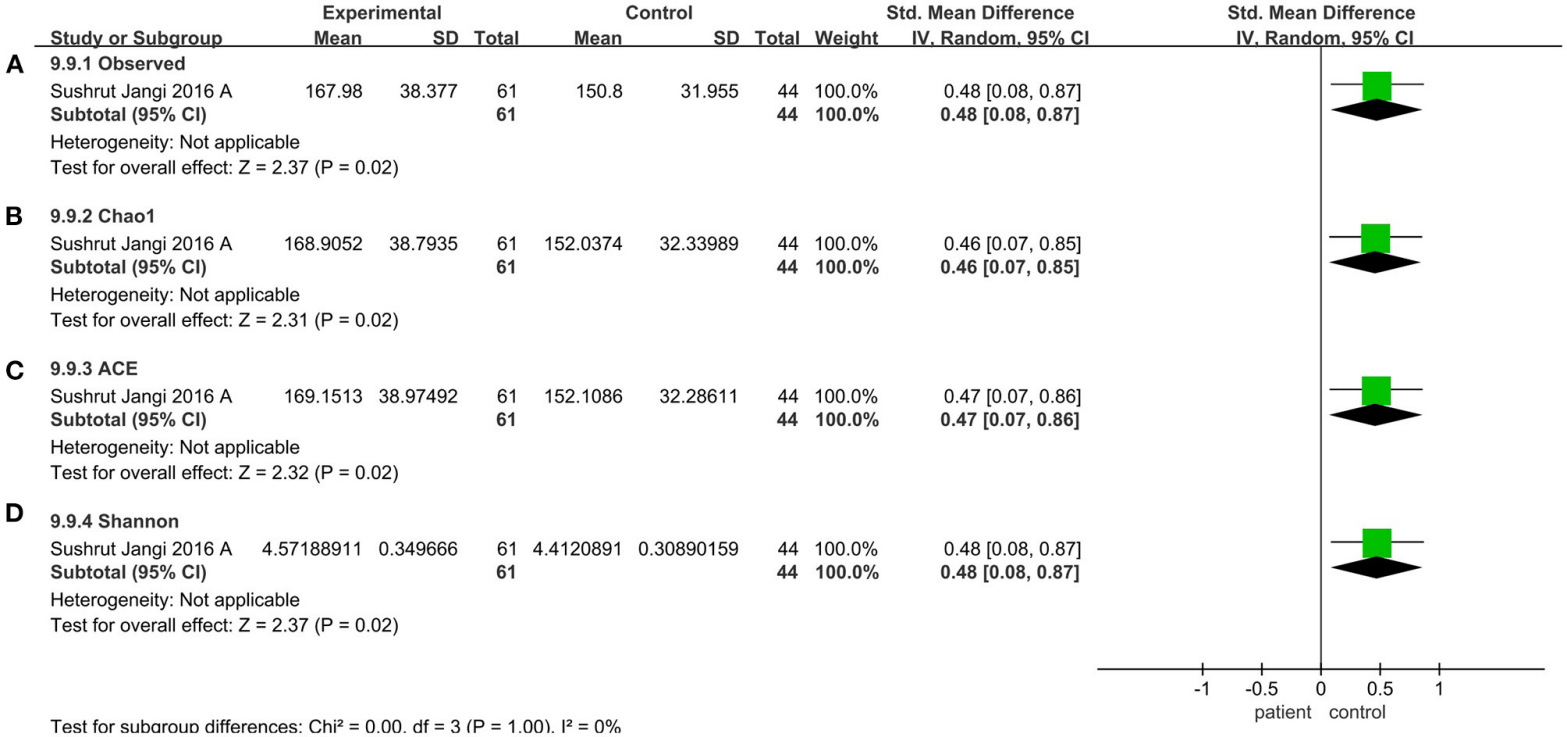
Diversity meta-analyses for the V3-V5 sequencing region. Forest plot for the observed species **(A)**, Chao1 **(B)**, ACE **(C)**, and Shannon **(D)**. Observed, observed species; SMD, standardized mean difference; SD, standard deviation.

V4 region sequencing was used in 29 studies. Compared to the control, the pooled estimate showed a decrease but no significant difference, including Shannon (SMD = −0.03; 95%CI, 0.22 to 0.17, *P* = 0.79, *I*^2^ = 76%) (see Supplementary Figure 5) and Simpson (SMD = −0.06; 95%CI, −0.24 to 0.12, *P* = 0.50, *I*^2^ = 71%) (see Supplementary Figure 6). But when we considered the V3-V5 sequencing region, there was a significant decrease in the patient group compared to the control group (observed species SMD = 0.48; 95%CI, 0.08 to 0.87; *P* = 0.02; Chao1 SMD = 0.46; 95%CI, 0.07 to 0.85; *P* = 0.02; ACE SMD = 0.47; 95%CI, 0.07 to 0.86; *P* = 0.02; Shannon SMD = 0.48; 95%CI, 0.08 to 0.87; *P* = 0.02) (Figure 7).

### Alpha Diversity Could Be an Indicator for Predicting AD, Schizophrenia, and MS

**Figure 8:**
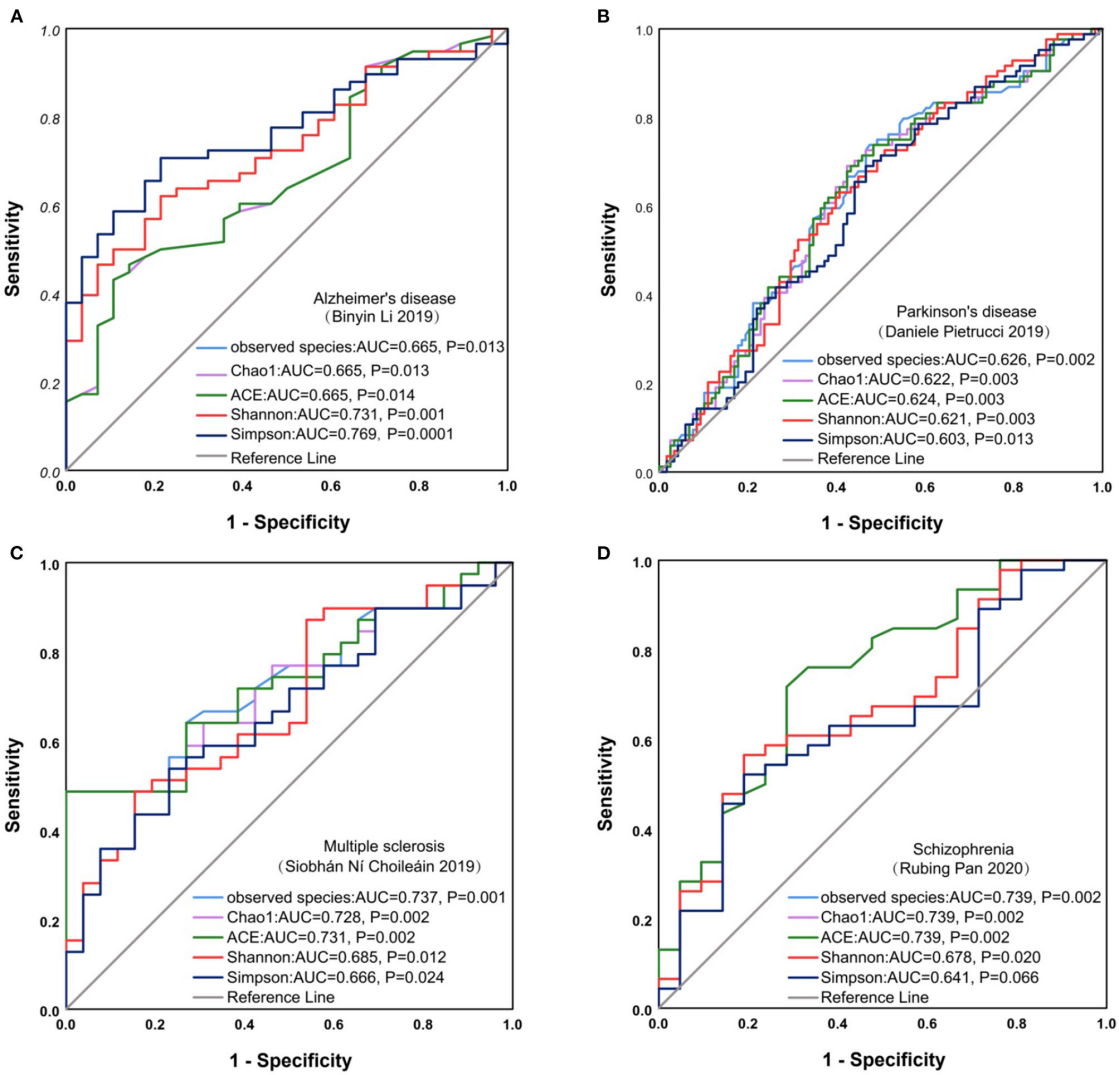
ROC curve analysis on neurological diseases. ROC curve for Alzheimer's disease **(A)**, Parkinson's disease **(B)**, Multiple sclerosis **(C)**, Schizophrenia **(D)**. ROC, receiver operating characteristic; AUC, area under the curve.

To furtherly investigate the relationship between the microbiota diversity and neurological diseases, we plotted the ROC curve of alpha diversity indices for AD, Parkinson's disease, schizophrenia, MS, and anorexia nervosa. Results from ROC curves illustrated that alpha diversity, such as Simpson, observed species, Chao1, and ACE index, based on the criterion of AUC >0.7, had a high potential to predict the AD (Shannon, AUC = 0.731, *P* = 0.001; Simpson, AUC = 0.769, *P* = 0.0001), MS (observed species, AUC = 0.737, *P* = 0.001; Chao1, AUC = 0.728, *P* = 0.002; ACE, AUC = 0.731, *P* = 0.002), schizophrenia (observed species, AUC = 0.739, *P* = 0.002; Chao1, AUC = 0.739, *P* = 0.002; ACE, AUC = 0.739, *P* = 0.002) (Figure 8), but not for the other neurological diseases (Supplementary Figure 7).

## Discussion

So far, there has not been a systematic review has comprehensively revealed the association between the gut microbiota and neurological diseases, and previous studies have been limited to individual diseases (Xu et al., 2019; Fu et al., 2020; Tuddenham et al., 2020; Shen et al., 2021; Zheng et al., 2021). Alpha diversity, as an important index to describe the characteristics of the gut microbiota, was considered to be highly related to the occurrence and development of many diseases. It was expected to decrease in disturbed systems, and low diversity is related to disease status and poor health, that is, dysbiosis (Reese and Dunn, 2018; Nuzum et al., 2020; Freidin et al., 2021). In this study, we focused on exploring whether the alpha diversity of the gut microbiota could be used as a predictor of neurological diseases, which is very important and helpful for the early diagnosis of these diseases that seriously affect the quality of life of patients.

This study systematically evaluated the relationship between the alpha diversity of the gut microbiota and neurological diseases, based on 2758 samples from China (Zhang et al., 2013; Li et al., 2019; Liu et al., 2019; Dan et al., 2020; Cao et al., 2021), Turkey (Safak et al., 2020), Italy (Coretti et al., 2018; Pietrucci et al., 2019), Germany (Mack et al., 2016; Weis et al., 2019), India (Pulikkan et al., 2018), Japan, America (Jangi, 2016; Nguyen et al., 2019; Choileáin et al., 2020), Korea, and Ecuador. Overall analysis was carried out and no significant differences in alpha diversity were observed between patients and controls. To evaluate the impact of disease classification on the gut microbiota, the subgroup analyses were performed on 24 studies, and alpha diversity showed a significant increase in anorexia nervosa, while a decrease in Parkinson's disease, compared to controls. These results demonstrated that alpha diversity of the gut microbiota could be a potential biomarker for predicting anorexia nervosa and Parkinson's disease. Previous studies have found similar results in the meta-analyses of psychiatric disorders, and Parkinson's disease (Nikolova et al., 2021; Plassais et al., 2021; McGuinness et al., 2022). Considering that different diseases have different pathological processes, the relationship between gut microbiota and disease progression may be more complex than just altered alpha diversity (Nuzum et al., 2020).

Furthermore, the ROC curve analysis showed that alpha diversity was able to diagnose AD, schizophrenia, and MS. These results demonstrate dysregulation and alteration in gut microbiota in AD, schizophrenia, and MS, which had been confirmed in the previous studies (Murray et al., 2021; Cantoni et al., 2022). Dysregulation of gut microbiota could facilitate the occurrence and development of neurological diseases, in which the “gut microbiota-brain” axis might play an important role (Almeida et al., 2020). Through the intestinal barriers, modulation of afferent sensory nerves, neurotransmitters, and finally regulation of bacterial metabolites, the biochemical signaling was sent from the gastrointestinal tract to the central nervous system (CNS) (Arneth, 2018; Vindegaard et al., 2021). By the “gut microbiota-brain” axis, the gut microbiota could regulate the secretion of serotonin (5-hydroxytryptamine, 5-HT) (Evans et al., 2013; Ghaisas et al., 2016), brain-derived nerve neurotransmitters such as brain-derived neurotrophic factor (BDNF) (Keightley et al., 2015), and gamma-aminobutyric acid (GABA) (Aho et al., 2019; Averina et al., 2020) and then act on the host nerves to produce excitatory or inhibitory effects on the CNS. In this sense, understanding alterations in microbial diversity and microbiota-host interactions will contribute to the development of microbiota-targeted interventions for AD, schizophrenia, and MS.

Nonetheless, our ultimate understanding of alpha diversity requires more than just measuring it, and we also need background data for thoughtful comparisons (Shade, 2017). So we should consider some potential confounding factors that contributed to microbial diversity, for example, the extrinsic factors (for c-section, non-breastfeeding, environment, lifestyle (Barandouzi et al., 2020), infection exposure, diet (Davis et al., 2017; Taylor et al., 2020), antibiotic use (Heiss and Olofsson, 2019), and age (Annalisa et al., 2014; Qian et al., 2020) and intrinsic factors (such as metabolite, immunity, hormone, and genetic background). They might affect the composition of the gut microbiota, which, in turn, affects the development of the individual. After we adjusted some variables, including sex, age, BMI, geographical region (east/west), hypervariable region sequenced, platform, and instrument, alpha diversity did not show a significant association with those neurodegeneration or neuropsychiatric diseases, which indicated that it might not be an all-round indicator for the whole neurological diseases.

The 16S rRNA gene amplified sequencing technology had become the main method for the identification and quantification of human-resident bacteria, due to its exceptional increases in numbers of reads and the lower associated cost (Weinstock, 2012; Sanada et al., 2020). We restricted the sequencing instruments and the sequencing regions in the studies to explore their impact on the diversity of the gut microbiota. When instrument Illumina HiSeq 2500 was restricted, alpha diversity was significantly decreased compared to the control groups. But when Illumina HiSeq 2000 and the V3-V5 sequencing region were restricted, alpha diversity was significantly increased. We surmise that some rare species could be lost during the sampling location and targeted amplicon sequencing of 16S rRNA gene amplified sequencing (including amplified fragment, and sequencing platforms), resulted in an underestimation of species richness (Finotello et al., 2018; Cheung et al., 2019; Lodovico et al., 2021), which has a certain influence on the gut microbiota (Lodovico et al., 2021).

There are limitations to our systematic review. Firstly, in our review, it's still missing in the great cohorts study of neurological disease and alpha-diversity. Secondly, we did not obtain all demographic data (age, sex, BMI, cigarette smoking, breastfeeding methods for early babies) and basic characteristics of the disease (including diagnostic criteria, and medication use) related to the subjects in all included studies. Some medications like antidepressants had been shown to have antibacterial effects (Vila et al., 2020). Thirdly, diet affects the gut microbiota, which, in turn, affects the health or disease state of the body. Different diets also contribute to bacterial diversity (David et al., 2014; Jiang et al., 2015). Furthermore, we only focused on the changes in the microbiota itself, and the relationship between the changes in the microbiota and other biomarkers, such as blood biomarkers, may also be an important orientation for future research (Vindegaard et al., 2021).

## Conclusions

In conclusion, our review summarized the association between alpha diversity of the gut microbiota and neurological diseases. The alpha diversity of gut microbiota could be a promising predictor for AD, schizophrenia, and MS, but not for all neurological diseases. We should consider two key characteristics that can lead to inconsistencies between studies, the sequencing region and the instrument of sequencing. In the future, the measurement of diversity should serve as a starting point for further research into ecological mechanisms, and a comprehensive, multidisciplinary research agenda is needed to further clarify the relationship between the gut microbiota and host health.

## Data Availability Statement

The datasets generated for this study can be found in the Bioproject and SRA PRJNA489760, PRJNA513960, PRJNA510730, PRJEB11199, PRJNA450340, PRJNA355023, PRJNA422961, PRJEB29421, PRJNA496408, PRJNA559773, PBJEB30615, PRJEB34168, PRJNA321051, PRJEB26004, PRJNA642975, PRJNA174838, PRJNA453621, PRJNA534155, PRJNA615774, PRJNA628832, PRJNA662563, PRJDB7752, PRJNA678145, and PRJEB27306.

## Author Contributions

JH and PC conceived and designed the study and revised the manuscript. HL and LY supervised the manuscript. ZL and JZ participated in writing the manuscript, interpreting the results, and preparing the report for publication. LL, FL, QW, TL, and XY participated in data acquisition. All authors contributed to the article and approved the submitted version.

## Funding

This study was supported by the Regional Fund Project of National Natural Science Foundation of China (NSFC, 82060366, 82160385), National Natural Science Foundation of China Youth Science Foundation project (NSFC, 82002134), and Natural Science Foundation of Guangxi (2018GXNSFAA050099).

## Conflict of Interest

The authors declare that the research was conducted in the absence of any commercial or financial relationships that could be construed as a potential conflict of interest.

## Publisher's Note

All claims expressed in this article are solely those of the authors and do not necessarily represent those of their affiliated organizations, or those of the publisher, the editors and the reviewers. Any product that may be evaluated in this article, or claim that may be made by its manufacturer, is not guaranteed or endorsed by the publisher.
