# Supplementary material for: Differences in Alpha Diversity of Gut Microbiota in Neurological Diseases
Source: Front Neurosci. 2022 Jun 28;16:879318. doi: 10.3389/fnins.2022.879318 (PMC9274120; doi:10.3389/fnins.2022.879318)
Supplement: Supplementary file 1 [file Table_1.DOCX]

Supplementary Material

# Supplementary Tables

**Table 1. Characteristics of studies included in PubMed**

| Author, year, country, title | Bioproject accession | Study design | Alpha diversity in patients and health controls |
| --- | --- | --- | --- |
| Binyin Li, 2019, China  Mild cognitive impairment has similar alterations as Alzheimer's disease in gut microbiota | **PRJNA489760** | cross-sectional study, fecal samples,  Alzheimer’s disease (AD) patients 30, mild cognitive impairment (MCI) patients 30, healthy controls 30,  V3-V4 region, Illumina MiSeq | ACE, Chao1, Shannon, Simpson No significant differences |
| Birol Şafak, 2019, Turkey  The gut microbiome in epilepsy | **PRJNA513960** | cross-sectional study, fecal samples,  epilepsy patients 30, healthy controls 10,  V3-V4 region, Illumina MiSeq | ND |
| Daniele Pietrucci, 2019, Italy  Dysbiosis of gut microbiota in a selected population of Parkinson's patients | **PRJNA510730** | cross-sectional study, fecal samples,  Parkinson's disease patients 80, healthy controls 72,  V3-V4 region, Illumina MiSeq | Chao1, Shannon, Simpson No significant differences |
| Isabelle Mack, 2016, Germany  Weight gain in anorexia nervosa does not ameliorate the faecal microbiota, branched chain fatty acid profiles, and gastrointestinal complaints | **PRJEB11199** | cohort study, fecal samples,  Anorexia nervosa (AN) patients 99, healthy controls 59,  V4 region, Illumina Miseq | observed species and Chao1 and Shannon index was significantly increased in increased AN after weight gain |
| Jessica D. Forbes, 2018, Canada  A comparative study of the gut microbiota in immune-mediated inflammatory diseases—does a common dysbiosis exist? | **PRJNA450340** | cohort study, fecal samples,  multiple sclerosis (MS) patients 19, healthy controls 23,  V4 region, Illumina Miseq | Chao1, ACE, Shannon, Simpson the Shannon and Simpson index was significantly decreased in MS patients |
| Joby Pulikkan, 2018, India  Gut Microbial Dysbiosis in Indian Children with Autism Spectrum Disorders | **PRJNA355023** | cross-sectional study, fecal samples,  autism spectrum disorder (ASD) patients 30, healthy controls 24,  V3 region, NextSeq 500 | observed species, Shannon, Phylogenetic diversity (PD) No significant differences |
| Junli Gong, 2018, China  Lack of short-chain fatty acids and overgrowth of opportunistic pathogens define dysbiosis of neuromyelitis optica spectrum disorders: A Chinese pilot study | **PRJNA422961** | cross-sectional study, fecal samples,  neuromyelitis optica spectrum disorders (NMOSD) patients 82, healthy controls 54,  ND, Illumina HiSeq 2500 | observed species, ACE, Chao1, Simpson, Shannon, Coverage  No significant differences |
| Lorena Coretti, 2018, Italy,  Gut Microbiota Features in Young Children With Autism Spectrum Disorders | **PRJEB29421** | Cross-sectional study, fecal samples,  autism spectrum disorder (ASD) patients 11, healthy controls 14,  V3-V4 region, Illumina Miseq | ASD significant higher in OTUs and Shannon index compare to control |
| Ping Liu, 2019, China,  Altered microbiomes distinguish Alzheimer’ s disease from amnestic mild cog-nitive impairment and health in a Chinese cohort | **PRJNA496408** | cohort study, fecal samples,  Alzheimer’ s disease (AD) patients 33, mild cognitive impairment (MCI) patients 32, healthy controls 32,  V3-V4 region, Illumina MiSeq | AD had significantly decreased in Shannon and Simpson but no significant differences in ACE and Chao 1 |
| Rubing Pan, 2020, China  Analysis of the diversity of intestinal microbiome and its potential value as a biomarker in patients with schizophrenia: A cohort study | **PRJNA559773** | cohort study, fecal samples,  symptomatic remission (rSCZ) patients 29, acute episode of SCZ (aSCZ) patients 29, healthy controls 29,  V3-V4 region, Illumina HiSeq 2000 | observed species, Shannon, Simpson, ACE, Chao, and PD No significant differences |
| Severin Weis, 2019, Germany,  Effect of Parkinson's disease and related medications on the composition of the fecal bacterial microbiota | **PRJEB30615** | Cross-sectional cohort study, fecal samples,  Parkinson's disease patients 34, healthy controls 25,  V4-V5 region, Illumina MiSeq | observed species and Chao1 decrease in Parkinson's disease but Shannon and Simpson metrics did not show significant differences between Parkinson's disease and control |
| Siobhán Ní Choileáin, 2019, USA, CXCR3+ T cells in multiple sclerosis correlate with reduced diversity of the gut microbiome | **PRJEB34168** | Cross-sectional cohort study, fecal samples,  Multiple sclerosis (MS) patients 26, healthy controls 39, V4 region, Illumina MiSeq | Shannon index was reduced in MS |
| Sushrut Jangi, 2016, USA,  Alterations of the human gut microbiome in multiple sclerosis | **PRJNA321051** | cross-sectional study**,** fecal samples,  Multiple sclerosis (MS) patients 60, healthy controls 43  V4 region, Illumina MiSeq,  Or V3-V5 region, 454 GS FLX | Shannon No significant differences |
| Tanya T. Nguyen，2019, USA, Differences in gut microbiome composition between persons with chronic schizophrenia and healthy comparison subjects | **PRJEB26004** | cross-sectional study, fecal samples,  Schizophrenia patients 25, Non-psychiatric Comparison 25, V4 region, Illumina MiSeq | observed species, Chao1, Shannon and Simpson No significant differences |
| Xia Cao, 2021, China,  Dysbiotic Gut Microbiota and Dysregulation of Cytokine Profile in Children and Teens With Autism Spectrum Disorder | **PRJNA642975** | cross-sectional study, fecal samples,  autism spectrum disorder (ASD) patients 45, healthy controls 41,  V4 region, Illumina HiSeq 2500 | An increase in a diversity (Shannon) among ASD group individuals |
| Zhigang Zhang, 2013, China  Large-scale survey of gut microbiota associated with MHE Via 16S rRNA-based pyrosequencing | **PRJNA174838** | cross-sectional study, fecal samples Minimal hepatic encephalopathy (MHE) patients 26, patients with cirrhosis without MHE (N group) 25, healthy controls 26, V2 region, 454 Life Sciences Genome Sequencer FLX instrument (Roche) | The results found a tendency toward a reduction in diversity (Chao1 and PD) (lower in the MHE and healthy controls; higher in the N group) |
| Zhou Dan, 2020, China  Altered gut microbial profile is associated with abnormal metabolism activity of Autism Spectrum Disorder | **PRJNA453621** | cross-sectional study, fecal samples Autistic Spectrum Disorder (ASD) patients 143, typically developing (TD) individuals 143 V4 region, Illumina Hiseq 2500 | PD and Shannon index was no significant differences between ASD and TD |

**Abbreviations:** ACE – abundance-based coverage estimator; ND - not described; OUT - Operational Taxonomic Units; PD - Phylogenetic Diversity;

**Table 2. Characteristics of studies not included.**

| Title, author, year, country | Bioproject accession | Study design | Results: Alpha diversity in patients/controls | Limitations/Potential bias |
| --- | --- | --- | --- | --- |
| Jun Chen, USA  Multiple sclerosis patients have a distinct gut microbiota compared to healthy controls | **PRJNA335855** | Stool samples RRMS 31, HCs 36, V3–V5 region | Chao1, Simpson, ACE, Shannon  no significant differences | ND |
| Congfu Huang, 2019, China  Distinct Gut Microbiota Composition and Functional Category in Children With Cerebral Palsy and Epilepsy | **PRJNA530084** | Cross-sectional cohort study, Fecal Sample, 25 CPE, 21 HC, V3-V4, Illumina MiSeq | OTUs significantly higher in CPE | lack of a large number of patients with epilepsy |
| Jia Yin, 2015, China,  Dysbiosis of Gut Microbiota With Reduced Trimethylamine‐N‐Oxide Level in Patients With Large‐Artery Atherosclerotic Stroke or Transient Ischemic Attack | **PRJEB9365** | case-control study, fecal samples, 141 Patients; 94 HC, V4, Illumina MiSeq | Chao1, observed species, Shannon significantly higher in TIA | ND |
| Valerio Iebba, 2018, Italy  Combining amplicon sequencing and metabolomics in cirrhotic patients highlights distinctive microbiota features involved in bacterial translocation, systemic inflammation and hepatic encephalopathy | **PRJNA471972** | Cross-sectional cohort study, Fecal samples, 89 cirrhotic patients; 20 HC, V3-V4, Illumina MiSeq | ND | ND |
