# Supplementary material for: Differences in Alpha Diversity of Gut Microbiota in Neurological Diseases
Source: Front Neurosci. 2022 Jun 28;16:879318. doi: 10.3389/fnins.2022.879318 (PMC9274120; doi:10.3389/fnins.2022.879318)
Supplement: Supplementary file 2 [file Image_1.pdf]

Supplementary Material

2 Supplementary Figures

Study or Subgroup	Patient			Control			Weight	Std. Mean Difference
	Mean	SD	Total	Mean	SD	Total		IV, Random, 95% CI
Binyin Li 2019 A	3.711428	0.347505	30	3.511487	0.290183	30	3.1%	0.62 [0.10, 1.14]
Binyin Li 2019 B	3.831395	0.298571	30	3.511487	0.290183	30	3.0%	1.07 [0.53, 1.62]
Birol Şafak 2019	4.399529	0.611401	30	4.666566	0.189426	10	2.2%	-0.48 [-1.21, 0.24]
China Beijing 2019	2.351041	0.46533	4	3.038463	0.494312	4	0.7%	-1.25 [-2.87, 0.38]
China Chongqing 2020	3.453914	0.562192	60	3.34265	0.620901	60	3.9%	0.19 [-0.17, 0.55]
China Zhejiang 2020	3.542414	0.298978	12	3.459612	0.313608	15	2.1%	0.26 [-0.50, 1.02]
Daniele Pietrucci 2019	4.951357	0.409979	118	5.110657	0.336797	84	4.3%	-0.42 [-0.70, -0.13]
India Mangalore 2020 A	3.195441	0.486605	22	3.053792	0.540529	36	3.0%	0.27 [-0.26, 0.80]
India Mangalore 2020 B	3.24469	0.547673	17	3.053792	0.540529	36	2.8%	0.35 [-0.23, 0.93]
Isabelle Mack 2016	3.640144	0.579847	99	3.422748	0.611061	59	4.1%	0.37 [0.04, 0.69]
Japan 2020 A	4.347062	0.622076	21	4.555096	0.406549	20	2.6%	-0.39 [-1.00, 0.23]
Japan 2020 B	4.545696	0.360368	62	4.555096	0.406549	20	3.1%	-0.03 [-0.53, 0.48]
Japan 2020 C	4.618774	0.304876	15	4.555096	0.406549	20	2.4%	0.17 [-0.50, 0.84]
Jessica D Forbes 2018	4.050203	0.472551	40	4.272491	0.339819	47	3.5%	-0.54 [-0.97, -0.11]
Joby Pulikkan 2018	2.718596	0.516922	30	2.759158	0.529601	24	3.0%	-0.08 [-0.61, 0.46]
JunliGong 2018	0.236486	0.330575	85	0.243885	0.334119	54	4.0%	-0.02 [-0.36, 0.32]
Korea2020	4.133171	0.480901	63	4.119327	0.488683	57	3.9%	0.03 [-0.33, 0.39]
Lorena Coretti 2018	1.338357	0.297897	11	1.490613	0.142387	14	1.9%	-0.66 [-1.47, 0.16]
Ping Liu 2019 A	3.70589	0.687732	33	4.015573	0.58777	28	3.1%	-0.47 [-0.99, 0.04]
Ping Liu 2019 B	4.23945	0.368306	32	4.015573	0.58777	28	3.1%	0.46 [-0.06, 0.97]
Rubing Pan 2020 A	4.134239	0.311993	24	3.769634	0.572184	23	2.7%	0.78 [0.19, 1.38]
Rubing Pan 2020 B	4.070436	0.405269	25	3.769634	0.572184	23	2.8%	0.60 [0.02, 1.18]
Severin Weis 2019	4.513287	0.454397	156	4.596867	0.364791	100	4.4%	-0.20 [-0.45, 0.05]
Siobhán Ní Choileáin 2019	3.418199	0.460231	26	3.743524	0.482043	39	3.1%	-0.68 [-1.19, -0.17]
South America Ecuador 2019	3.671945	0.498633	27	3.596494	0.446585	31	3.1%	0.16 [-0.36, 0.67]
Sushrut Jangi 2016 A	4.571889	0.349666	61	4.412089	0.308902	44	3.7%	0.48 [0.08, 0.87]
Sushrut Jangi 2016 B	3.622959	0.459037	61	3.604435	0.454245	44	3.7%	0.04 [-0.35, 0.43]
Tanya T. Nguyen 2019	3.378607	0.680134	73	3.58207	0.525929	73	4.1%	-0.33 [-0.66, -0.01]
Xia Cao 2021	3.891775	0.212122	8	4.020752	0.237221	41	2.1%	-0.54 [-1.31, 0.22]
Zhigang Zhang 2013 A	2.252523	0.675718	25	2.34431	0.618806	26	2.9%	-0.14 [-0.69, 0.41]
Zhigang Zhang 2013 B	2.196717	0.463269	26	2.34431	0.618806	26	2.9%	-0.27 [-0.81, 0.28]
Zhou Dan 2020	1.31497	0.311741	143	1.393983	0.31826	143	4.5%	-0.25 [-0.48, -0.02]
**Total (95% CI)**			**1469**			**1289**	**100.0%**	**0.00 [-0.14, 0.15]**

Heterogeneity: $Tau^2 = 0.10$; $Chi^2 = 94.83$, $df = 31$ ($P < 0.00001$); $I^2 = 67\%$

Test for overall effect: $Z = 0.03$ ($P = 0.98$)

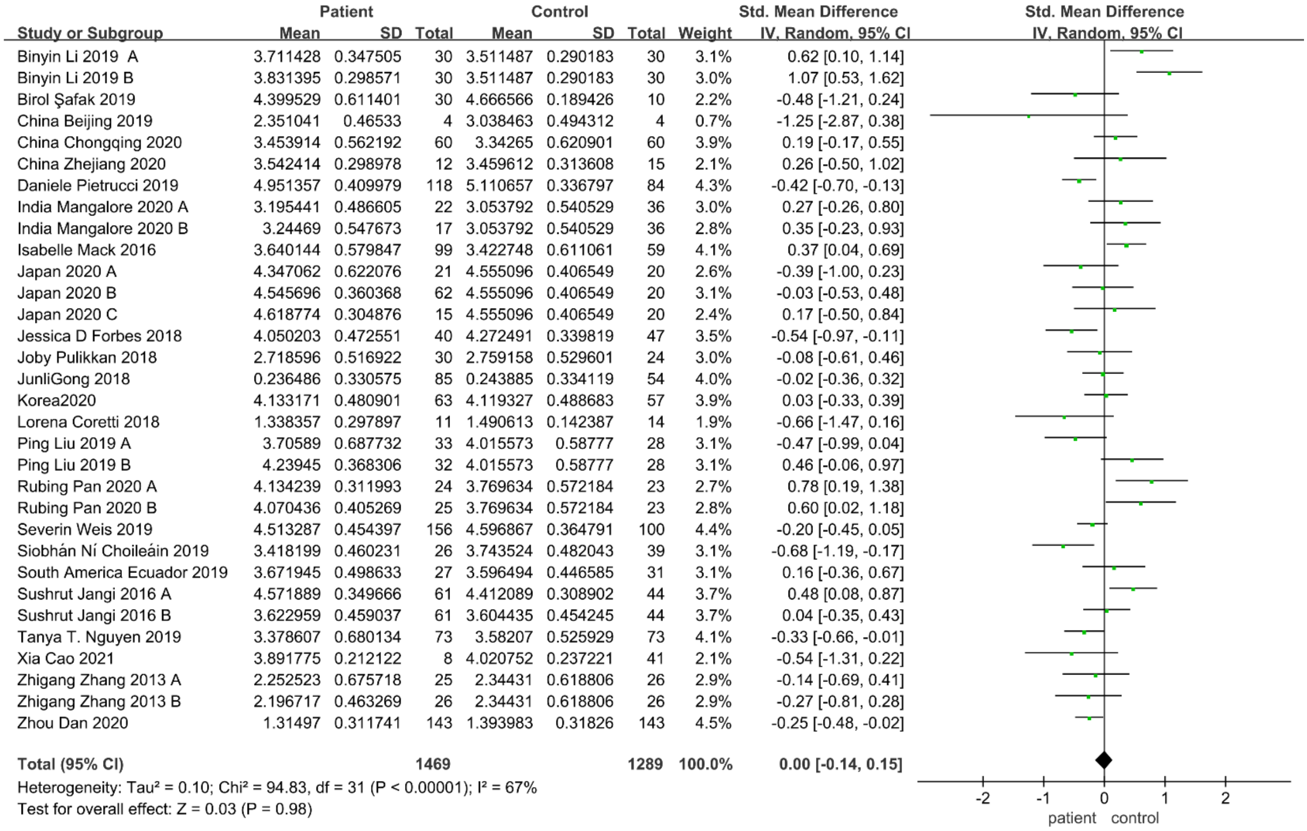

Supplementary Figure 1. Forest plots of alpha diversity in the gut microbiome of patients with neurological diseases compared with healthy controls. Abbreviations: SMD, standardized mean difference; SD, standard deviation.

Study or Subgroup	Patient Mean	Patient SD	Patient Total	Control Mean	Control SD	Control Total	Weight	Std. Mean Difference IV, Random, 95% CI
**2.4.1 Alzheimer's disease**								
Binyin Li 2019 A	3.711428	0.347505	30	3.511487	0.290183	30	3.1%	0.62 [0.10, 1.14]
Binyin Li 2019 B	3.831395	0.298571	30	3.511487	0.290183	30	3.0%	1.07 [0.53, 1.62]
Ping Liu 2019 A	3.70589	0.687732	33	4.015573	0.58777	28	3.1%	-0.47 [-0.99, 0.04]
Ping Liu 2019 B	4.23945	0.368306	32	4.015573	0.58777	28	3.1%	0.46 [-0.06, 0.97]
**Subtotal (95% CI)**			**125**			**116**	**12.2%**	**0.41 [-0.22, 1.05]**
Heterogeneity: Tau² = 0.35; Chi² = 17.85, df = 3 (P = 0.0005); I² = 83%								
Test for overall effect: Z = 1.28 (P = 0.20)								
**2.4.2 Anorexia nervosa**								
Isabelle Mack 2016	3.640144	0.579847	99	3.422748	0.611061	59	4.1%	0.37 [0.04, 0.69]
**Subtotal (95% CI)**			**99**			**59**	**4.1%**	**0.37 [0.04, 0.69]**
Heterogeneity: Not applicable								
Test for overall effect: Z = 2.21 (P = 0.03)								
**2.4.3 Autism Spectrum Disorder**								
China Chongqing 2020	3.453914	0.562192	60	3.34265	0.620901	60	3.9%	0.19 [-0.17, 0.55]
Joby Pulikkan 2018	2.718596	0.516922	30	2.759158	0.529601	24	3.0%	-0.08 [-0.61, 0.46]
Lorena Coretti 2018	1.338357	0.297897	11	1.490613	0.142387	14	1.9%	-0.66 [-1.47, 0.16]
South America Ecuador 2019	3.671945	0.498633	27	3.596494	0.446585	31	3.1%	0.16 [-0.36, 0.67]
Xia Cao 2021	3.891775	0.212122	8	4.020752	0.237221	41	2.1%	-0.54 [-1.31, 0.22]
Zhou Dan 2020	1.31497	0.311741	143	1.393983	0.31826	143	4.5%	-0.25 [-0.48, -0.02]
**Subtotal (95% CI)**			**279**			**313**	**18.5%**	**-0.12 [-0.36, 0.12]**
Heterogeneity: Tau² = 0.03; Chi² = 7.98, df = 5 (P = 0.16); I² = 37%								
Test for overall effect: Z = 0.97 (P = 0.33)								
**2.4.4 epilepsy**								
Birol Şafak 2019	4.399529	0.611401	30	4.666566	0.189426	10	2.2%	-0.48 [-1.21, 0.24]
**Subtotal (95% CI)**			**30**			**10**	**2.2%**	**-0.48 [-1.21, 0.24]**
Heterogeneity: Not applicable								
Test for overall effect: Z = 1.31 (P = 0.19)								
**2.4.5 Hepatic encephalopathy**								
China Beijing 2019	2.351041	0.46533	4	3.038463	0.494312	4	0.7%	-1.25 [-2.87, 0.38]
Zhigang Zhang 2013 A	2.252523	0.675718	25	2.34431	0.618806	26	2.9%	-0.14 [-0.69, 0.41]
Zhigang Zhang 2013 B	2.196717	0.463269	26	2.34431	0.618806	26	2.9%	-0.27 [-0.81, 0.28]
**Subtotal (95% CI)**			**55**			**56**	**6.5%**	**-0.26 [-0.64, 0.12]**
Heterogeneity: Tau² = 0.00; Chi² = 1.59, df = 2 (P = 0.45); I² = 0%								
Test for overall effect: Z = 1.35 (P = 0.18)								
**2.4.6 Multiple sclerosis**								
China Zhejiang 2020	3.542414	0.298978	12	3.459612	0.313608	15	2.1%	0.26 [-0.50, 1.02]
Japan 2020 A	4.347062	0.622076	21	4.555096	0.406549	20	2.6%	-0.39 [-1.00, 0.23]
Japan 2020 B	4.545696	0.360368	62	4.555096	0.406549	20	3.1%	-0.03 [-0.53, 0.48]
Japan 2020 C	4.618774	0.304876	15	4.555096	0.406549	20	2.4%	0.17 [-0.50, 0.84]
Jessica D Forbes 2018	4.050203	0.472551	40	4.272491	0.339819	47	3.5%	-0.54 [-0.97, -0.11]
Siobhán Ní Choileáin 2019	3.418199	0.460231	26	3.743524	0.482043	39	3.1%	-0.68 [-1.19, -0.17]
Sushrut Jangi 2016 A	4.571889	0.349666	61	4.412089	0.308902	44	3.7%	0.48 [0.08, 0.87]
Sushrut Jangi 2016 B	3.622959	0.459037	61	3.604435	0.454245	44	3.7%	0.04 [-0.35, 0.43]
**Subtotal (95% CI)**			**298**			**249**	**24.4%**	**-0.09 [-0.40, 0.21]**
Heterogeneity: Tau² = 0.12; Chi² = 20.07, df = 7 (P = 0.005); I² = 65%								
Test for overall effect: Z = 0.59 (P = 0.55)								
**2.4.7 Optic neuromyelitis**								
India Mangalore 2020 A	3.195441	0.486605	22	3.053792	0.540529	36	3.0%	0.27 [-0.26, 0.80]
India Mangalore 2020 B	3.24469	0.547673	17	3.053792	0.540529	36	2.8%	0.35 [-0.23, 0.93]
JunliGong 2018	0.236486	0.330575	85	0.243885	0.334119	54	4.0%	-0.02 [-0.36, 0.32]
**Subtotal (95% CI)**			**124**			**126**	**9.8%**	**0.12 [-0.14, 0.38]**
Heterogeneity: Tau² = 0.00; Chi² = 1.55, df = 2 (P = 0.46); I² = 0%								
Test for overall effect: Z = 0.90 (P = 0.37)								
**2.4.8 Parkinson's Disease**								
Daniele Pietrucci 2019	4.951357	0.409979	118	5.110657	0.336797	84	4.3%	-0.42 [-0.70, -0.13]
Severin Weis 2019	4.513287	0.454397	156	4.596867	0.364791	100	4.4%	-0.20 [-0.45, 0.05]
**Subtotal (95% CI)**			**274**			**184**	**8.7%**	**-0.30 [-0.51, -0.08]**
Heterogeneity: Tau² = 0.01; Chi² = 1.28, df = 1 (P = 0.26); I² = 22%								
Test for overall effect: Z = 2.73 (P = 0.006)								
**2.4.9 Schizophrenia**								
Rubing Pan 2020 A	4.134239	0.311993	24	3.769634	0.572184	23	2.7%	0.78 [0.19, 1.38]
Rubing Pan 2020 B	4.070436	0.405269	25	3.769634	0.572184	23	2.8%	0.60 [0.02, 1.18]
Tanya T. Nguyen 2019	3.378607	0.680134	73	3.58207	0.525929	73	4.1%	-0.33 [-0.66, -0.01]
**Subtotal (95% CI)**			**122**			**119**	**9.6%**	**0.32 [-0.45, 1.09]**
Heterogeneity: Tau² = 0.40; Chi² = 14.56, df = 2 (P = 0.0007); I² = 86%								
Test for overall effect: Z = 0.81 (P = 0.42)								
**2.4.10 depression**								
Korea2020	4.133171	0.480901	63	4.119327	0.488683	57	3.9%	0.03 [-0.33, 0.39]
**Subtotal (95% CI)**			**63**			**57**	**3.9%**	**0.03 [-0.33, 0.39]**
Heterogeneity: Not applicable								
Test for overall effect: Z = 0.16 (P = 0.88)								
**Total (95% CI)**			**1469**			**1289**	**100.0%**	**0.00 [-0.14, 0.15]**
Heterogeneity: Tau² = 0.10; Chi² = 94.83, df = 31 (P < 0.00001); I² = 67%								
Test for overall effect: Z = 0.03 (P = 0.98)								
Test for subgroup differences: Chi² = 19.11, df = 9 (P = 0.02), I² = 52.9%								

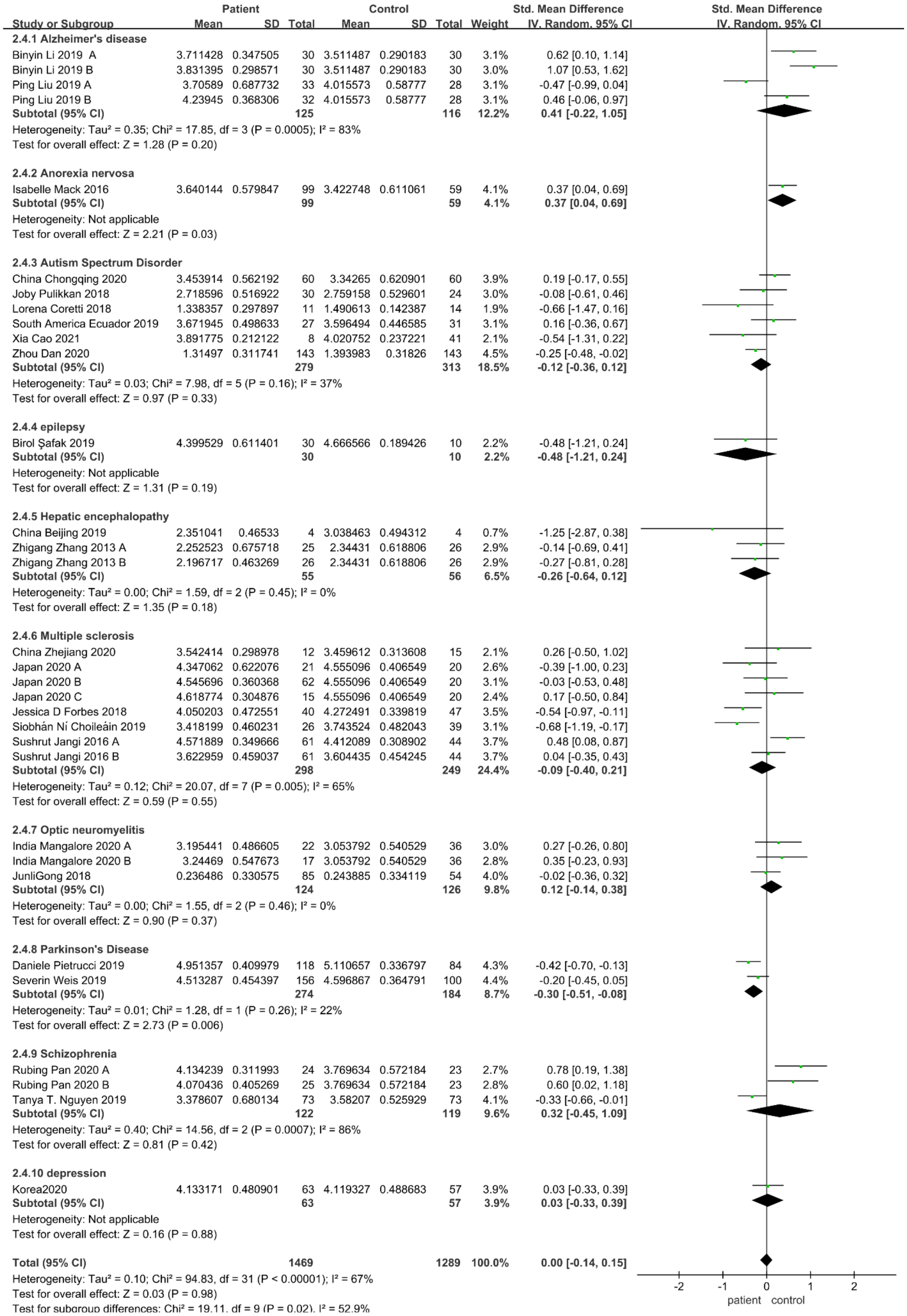

Supplementary Figure 2. Forest plot of the Shannon for the diseases. Abbreviations: SMD, standardized mean difference; SD, standard deviation.

Study or Subgroup	Patient Mean	Patient SD	Patient Total	Control Mean	Control SD	Control Total	Weight	Std. Mean Difference IV, Random, 95% CI
**12.4.1 Illumina MiSeq**								
Binyin Li 2019 A	3.71142757	0.347505	30	3.51148693	0.29018263	30	3.1%	0.62 [0.10, 1.14]
Binyin Li 2019 B	3.83139458	0.298571	30	3.51148693	0.29018263	30	3.0%	1.07 [0.53, 1.62]
Birol Şafak 2019	4.39952949	0.611401	30	4.66656552	0.18942592	10	2.2%	-0.48 [-1.21, 0.24]
China Beijing 2019	2.3510412	0.46533	4	3.03846286	0.4943121	4	0.7%	-1.25 [-2.87, 0.38]
China Chongqing 2020	3.45391444	0.562192	60	3.34264993	0.62090114	60	3.9%	0.19 [-0.17, 0.55]
China Zhejiang 2020	3.54241383	0.298978	12	3.45961182	0.31360839	15	2.1%	0.26 [-0.50, 1.02]
Daniele Pietrucci 2019	4.95135726	0.409979	118	5.11065658	0.33679716	84	4.3%	-0.42 [-0.70, -0.13]
India Mangalore 2020 A	3.19544079	0.486605	22	3.05379196	0.54052913	36	3.0%	0.27 [-0.26, 0.80]
India Mangalore 2020 B	3.24468957	0.547673	17	3.05379196	0.54052913	36	2.8%	0.35 [-0.23, 0.93]
Isabelle Mack 2016	3.64014386	0.579847	99	3.42274765	0.61106109	59	4.1%	0.37 [0.04, 0.69]
Japan 2020 A	4.34706185	0.622076	21	4.55509584	0.40654921	20	2.6%	-0.39 [-1.00, 0.23]
Japan 2020 B	4.54569555	0.360368	62	4.55509584	0.40654921	20	3.1%	-0.03 [-0.53, 0.48]
Japan 2020 C	4.61877435	0.304876	15	4.55509584	0.40654921	20	2.4%	0.17 [-0.50, 0.84]
Jessica D Forbes 2018	4.05020292	0.472551	40	4.27249137	0.3398189	47	3.5%	-0.54 [-0.97, -0.11]
Lorena Coretti 2018	1.33835719	0.297897	11	1.49061268	0.14238701	14	1.9%	-0.66 [-1.47, 0.16]
Ping Liu 2019 A	3.70589022	0.687732	33	4.01557263	0.58776968	28	3.1%	-0.47 [-0.99, 0.04]
Ping Liu 2019 B	4.23944957	0.368306	32	4.01557263	0.58776968	28	3.1%	0.46 [-0.06, 0.97]
Severin Weis 2019	4.51328665	0.454397	156	4.59686694	0.36479055	100	4.4%	-0.20 [-0.45, 0.05]
Siobhán Ní Choileáin 2019	3.41819868	0.460231	26	3.7435243	0.48204265	39	3.1%	-0.68 [-1.19, -0.17]
South America Ecuador 2019	3.67194533	0.498633	27	3.59649407	0.44658535	31	3.1%	0.16 [-0.36, 0.67]
Sushrut Jangi 2016 B	3.62295946	0.459037	61	3.60443513	0.4542453	44	3.7%	0.04 [-0.35, 0.43]
Tanya T. Nguyen 2019	3.37860676	0.680134	73	3.58207035	0.52592878	73	4.1%	-0.33 [-0.66, -0.01]
**Subtotal (95% CI)**			**979**			**828**	**67.4%**	**-0.03 [-0.21, 0.16]**
Heterogeneity: Tau² = 0.13; Chi² = 70.35, df = 21 (P < 0.00001); I² = 70%								
Test for overall effect: Z = 0.27 (P = 0.78)								
**12.4.2 Illumina HiSeq 2500**								
JunliGong 2018	0.23648551	0.330575	85	0.24388512	0.33411923	54	4.0%	-0.02 [-0.36, 0.32]
Xia Cao 2021	3.89177459	0.212122	8	4.0207517	0.23722116	41	2.1%	-0.54 [-1.31, 0.22]
Zhou Dan 2020	1.31497006	0.311741	143	1.39398292	0.31825954	143	4.5%	-0.25 [-0.48, -0.02]
**Subtotal (95% CI)**			**236**			**238**	**10.6%**	**-0.20 [-0.39, -0.01]**
Heterogeneity: Tau² = 0.00; Chi² = 1.99, df = 2 (P = 0.37); I² = 0%								
Test for overall effect: Z = 2.10 (P = 0.04)								
**12.4.3 Illumina HiSeq 2000**								
Rubing Pan 2020 A	4.13423941	0.311993	24	3.76963409	0.57218401	23	2.7%	0.78 [0.19, 1.38]
Rubing Pan 2020 B	4.0704356	0.405269	25	3.76963409	0.57218401	23	2.8%	0.60 [0.02, 1.18]
**Subtotal (95% CI)**			**49**			**46**	**5.5%**	**0.69 [0.27, 1.10]**
Heterogeneity: Tau² = 0.00; Chi² = 0.18, df = 1 (P = 0.67); I² = 0%								
Test for overall effect: Z = 3.25 (P = 0.001)								
**12.4.4 Illumina iSeq 100**								
Korea2020	4.13317063	0.480901	63	4.1193269	0.4886829	57	3.9%	0.03 [-0.33, 0.39]
**Subtotal (95% CI)**			**63**			**57**	**3.9%**	**0.03 [-0.33, 0.39]**
Heterogeneity: Not applicable								
Test for overall effect: Z = 0.16 (P = 0.88)								
**12.4.5 NextSeq 500**								
Joby Pulikkan 2018	2.71859626	0.516922	30	2.75915782	0.52960067	24	3.0%	-0.08 [-0.61, 0.46]
**Subtotal (95% CI)**			**30**			**24**	**3.0%**	**-0.08 [-0.61, 0.46]**
Heterogeneity: Not applicable								
Test for overall effect: Z = 0.28 (P = 0.78)								
**12.4.6 454 GS FLX**								
Sushrut Jangi 2016 A	4.57188911	0.349666	61	4.4120891	0.30890159	44	3.7%	0.48 [0.08, 0.87]
Zhigang Zhang 2013 A	2.25252284	0.675718	25	2.34430996	0.6188058	26	2.9%	-0.14 [-0.69, 0.41]
Zhigang Zhang 2013 B	2.19671709	0.463269	26	2.34430996	0.6188058	26	2.9%	-0.27 [-0.81, 0.28]
**Subtotal (95% CI)**			**112**			**96**	**9.6%**	**0.06 [-0.43, 0.55]**
Heterogeneity: Tau² = 0.12; Chi² = 5.92, df = 2 (P = 0.05); I² = 66%								
Test for overall effect: Z = 0.22 (P = 0.82)								
**Total (95% CI)**			**1469**			**1289**	**100.0%**	**0.00 [-0.14, 0.15]**
Heterogeneity: Tau² = 0.10; Chi² = 94.83, df = 31 (P < 0.00001); I² = 67%								
Test for overall effect: Z = 0.03 (P = 0.98)								
Test for subgroup differences: Chi² = 14.94, df = 5 (P = 0.01), I² = 66.5%								

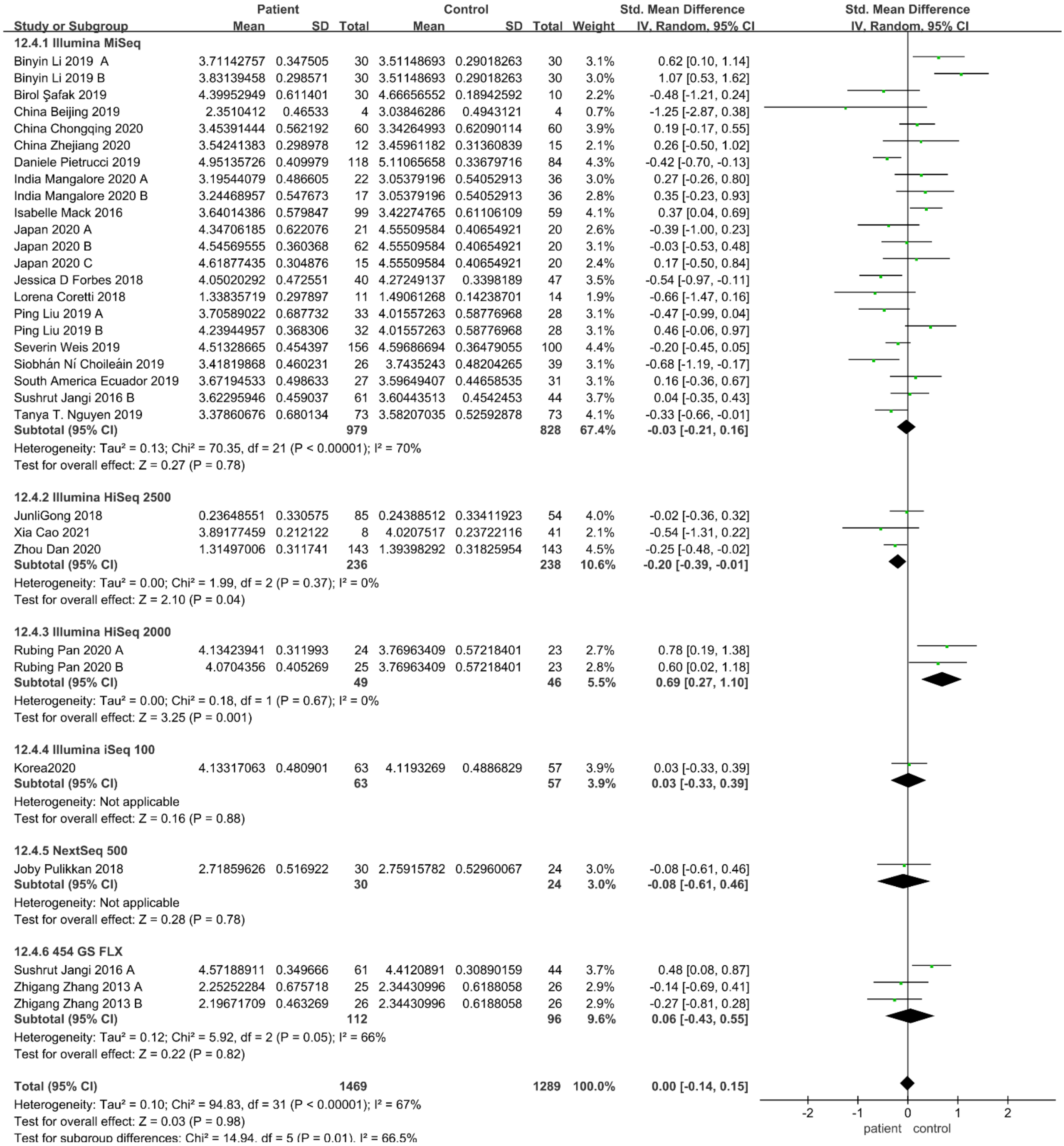

Supplementary Figure 3. Forest plot of the Shannon for the sequencing instrument.
Abbreviations: SMD, standardized mean difference; SD, standard deviation.

Study or Subgroup	Experimental Mean	Experimental SD	Experimental Total	Control Mean	Control SD	Control Total	Weight	Std. Mean Difference IV, Random, 95% CI
**12.5.1 Illumina MiSeq**								
Binyin Li 2019 A	0.96742	0.014119	30	0.960637	0.011909	30	3.1%	0.51 [-0.00, 1.03]
Binyin Li 2019 B	0.972584	0.011957	30	0.960637	0.011909	30	3.0%	0.99 [0.45, 1.53]
Birol Şafak 2019	0.978754	0.02182	30	0.988481	0.002289	10	2.2%	-0.50 [-1.22, 0.23]
China Beijing 2019	0.859121	0.067894	4	0.931986	0.03796	4	0.7%	-1.15 [-2.75, 0.44]
China Chongqing 2020	0.940466	0.036265	60	0.929537	0.066379	60	4.0%	0.20 [-0.16, 0.56]
China Zhejiang 2020	0.961754	0.011942	12	0.957659	0.013459	15	2.1%	0.31 [-0.45, 1.07]
Daniele Pietrucci 2019	0.98766	0.006604	118	0.98972	0.005154	84	4.4%	-0.34 [-0.62, -0.06]
India Mangalore 2020 A	0.90126	0.054238	22	0.878041	0.062693	36	3.0%	0.38 [-0.15, 0.92]
India Mangalore 2020 B	0.913744	0.042556	17	0.878041	0.062693	36	2.8%	0.62 [0.03, 1.21]
Isabelle Mack 2016	0.924276	0.079449	99	0.890296	0.106682	59	4.2%	0.37 [0.05, 0.70]
Japan 2020 A	0.96697	0.055824	21	0.980461	0.012497	20	2.7%	-0.32 [-0.94, 0.29]
Japan 2020 B	0.980814	0.009664	62	0.980461	0.012497	20	3.2%	0.03 [-0.47, 0.54]
Japan 2020 C	0.983946	0.007119	15	0.980461	0.012497	20	2.4%	0.32 [-0.35, 1.00]
Jessica D Forbes 2018	0.965733	0.023717	40	0.976369	0.008744	47	3.6%	-0.61 [-1.04, -0.18]
Lorena Coretti 2018	0.712727	0.104028	11	0.765714	0.041084	14	1.9%	-0.68 [-1.50, 0.13]
Ping Liu 2019 A	0.954275	0.038126	33	0.968519	0.027939	28	3.2%	-0.42 [-0.92, 0.09]
Ping Liu 2019 B	0.978002	0.010758	32	0.968519	0.027939	28	3.1%	0.45 [-0.06, 0.97]
Severin Weis 2019	0.980233	0.011797	156	0.982642	0.008301	100	4.6%	-0.23 [-0.48, 0.02]
Siobhán Ní Choileáin 2019	0.92643	0.041201	26	0.944311	0.034393	39	3.2%	-0.47 [-0.98, 0.03]
South America Ecuador 2019	0.942651	0.042346	27	0.939691	0.043555	31	3.1%	0.07 [-0.45, 0.58]
Sushrut Jangi 2016 B	0.895817	0.110787	73	0.921409	0.043338	73	4.2%	-0.30 [-0.63, 0.02]
Tanya T. Nguyen 2019	0.970678	0.010062	8	0.976781	0.005662	41	2.0%	-0.92 [-1.70, -0.14]
**Subtotal (95% CI)**			**926**			**825**	**66.8%**	**-0.03 [-0.22, 0.16]**
Heterogeneity: Tau² = 0.13; Chi² = 71.33, df = 21 (P < 0.00001); I² = 71%								
Test for overall effect: Z = 0.29 (P = 0.77)								
**12.5.2 Illumina HiSeq 2500**								
JunliGong 2018	0.171	0.2385	85	0.176	0.241	54	4.1%	-0.02 [-0.36, 0.32]
Xia Cao 2021	0.970678	0.010062	8	0.976781	0.005662	41	2.0%	-0.92 [-1.70, -0.14]
Zhou Dan 2020	0.652753	0.116871	143	0.676355	0.111834	143	4.7%	-0.21 [-0.44, 0.03]
**Subtotal (95% CI)**			**236**			**238**	**10.8%**	**-0.23 [-0.56, 0.09]**
Heterogeneity: Tau² = 0.04; Chi² = 4.34, df = 2 (P = 0.11); I² = 54%								
Test for overall effect: Z = 1.41 (P = 0.16)								
**12.5.3 Illumina HiSeq 2000**								
Rubing Pan 2020 A	0.977219	0.009476	24	0.964406	0.028404	23	2.8%	0.60 [0.01, 1.19]
Rubing Pan 2020 B	0.97458	0.014541	25	0.964406	0.028404	23	2.9%	0.45 [-0.12, 1.02]
**Subtotal (95% CI)**			**49**			**46**	**5.7%**	**0.52 [0.11, 0.93]**
Heterogeneity: Tau² = 0.00; Chi² = 0.13, df = 1 (P = 0.72); I² = 0%								
Test for overall effect: Z = 2.50 (P = 0.01)								
**12.5.4 Illumina iSeq 100**								
Korea2020	0.970616	0.020106	63	0.967437	0.025908	57	4.0%	0.14 [-0.22, 0.50]
**Subtotal (95% CI)**			**63**			**57**	**4.0%**	**0.14 [-0.22, 0.50]**
Heterogeneity: Not applicable								
Test for overall effect: Z = 0.75 (P = 0.45)								
**12.5.5 NextSeq 500**								
Joby Pulikkan 2018	0.836505	0.1186	30	0.849823	0.082381	24	3.0%	-0.13 [-0.66, 0.41]
**Subtotal (95% CI)**			**30**			**24**	**3.0%**	**-0.13 [-0.66, 0.41]**
Heterogeneity: Not applicable								
Test for overall effect: Z = 0.46 (P = 0.65)								
**12.5.6 454 GS FLX**								
Sushrut Jangi 2016 A	0.982433	0.009915	61	0.979635	0.008913	44	3.8%	0.29 [-0.10, 0.68]
Zhigang Zhang 2013 A	0.802544	0.170379	25	0.840322	0.110268	26	3.0%	-0.26 [-0.81, 0.29]
Zhigang Zhang 2013 B	0.831332	0.117622	26	0.840322	0.110268	26	3.0%	-0.08 [-0.62, 0.47]
**Subtotal (95% CI)**			**112**			**96**	**9.8%**	**0.03 [-0.30, 0.37]**
Heterogeneity: Tau² = 0.03; Chi² = 2.90, df = 2 (P = 0.23); I² = 31%								
Test for overall effect: Z = 0.20 (P = 0.84)								
**Total (95% CI)**			**1416**			**1286**	**100.0%**	**-0.01 [-0.16, 0.13]**
Heterogeneity: Tau² = 0.10; Chi² = 90.08, df = 31 (P < 0.00001); I² = 66%								
Test for overall effect: Z = 0.20 (P = 0.84)								
Test for subgroup differences: Chi² = 9.06, df = 5 (P = 0.11), I² = 44.8%								

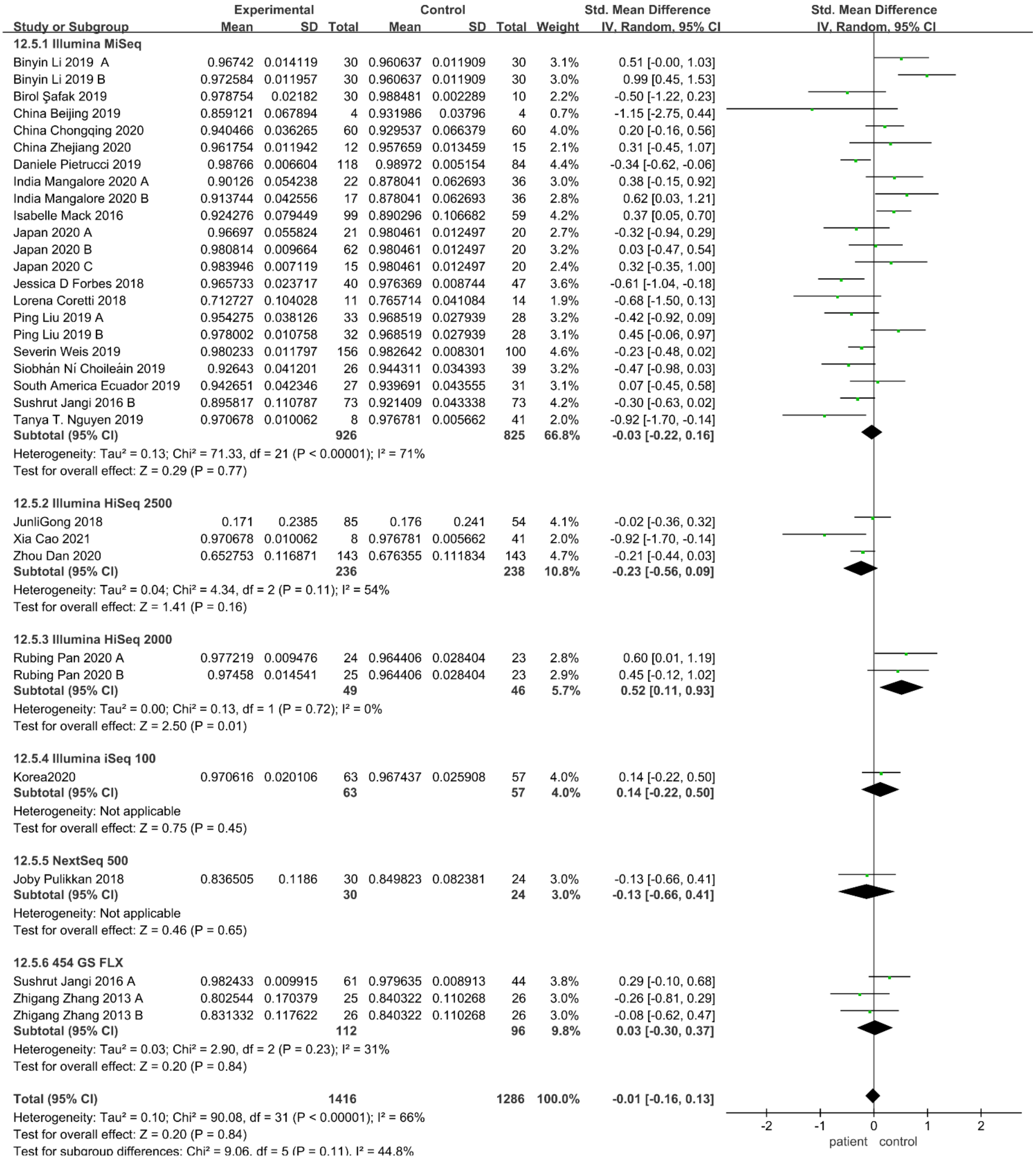

Supplementary Figure 4. Forest plot of the Simpson for the sequencing instrument.
Abbreviations: SMD, standardized mean difference; SD, standard deviation.

Study or Subgroup	Patient Mean	Patient SD	Patient Total	Control Mean	Control SD	Control Total	Weight	Std. Mean Difference IV, Random, 95% CI
**9.4.1 V2**								
Zhigang Zhang 2013 A	2.25252284	0.675718	25	2.34430996	0.6188058	26	4.4%	-0.14 [-0.69, 0.41]
Zhigang Zhang 2013 B	2.19671709	0.463269	26	2.34430996	0.6188058	26	4.4%	-0.27 [-0.81, 0.28]
**Subtotal (95% CI)**			**51**			**52**	**8.9%**	**-0.20 [-0.59, 0.18]**
Heterogeneity: Tau² = 0.00; Chi² = 0.10, df = 1 (P = 0.75); I² = 0%								
Test for overall effect: Z = 1.03 (P = 0.30)								
**9.4.2 V3**								
Joby Pulikkan 2018	2.71859626	0.516922	30	2.75915782	0.52960067	24	4.5%	-0.08 [-0.61, 0.46]
**Subtotal (95% CI)**			**30**			**24**	**4.5%**	**-0.08 [-0.61, 0.46]**
Heterogeneity: Not applicable								
Test for overall effect: Z = 0.28 (P = 0.78)								
**9.4.3 V3-V4**								
Binyin Li 2019 A	3.71142757	0.347505	30	3.51148693	0.29018263	30	4.6%	0.62 [0.10, 1.14]
Binyin Li 2019 B	3.83139458	0.298571	30	3.51148693	0.29018263	30	4.4%	1.07 [0.53, 1.62]
Birol Şafak 2019	4.39952949	0.611401	30	4.66656552	0.18942592	10	3.5%	-0.48 [-1.21, 0.24]
Daniele Pietrucci 2019	4.95135726	0.409979	118	5.11065658	0.33679716	84	5.9%	-0.42 [-0.70, -0.13]
Lorena Coretti 2018	1.33835719	0.297897	11	1.49061268	0.14238701	14	3.1%	-0.66 [-1.47, 0.16]
Ping Liu 2019 A	3.70589022	0.687732	33	4.01557263	0.58776968	28	4.6%	-0.47 [-0.99, 0.04]
Ping Liu 2019 B	4.23944957	0.368306	32	4.01557263	0.58776968	28	4.6%	0.46 [-0.06, 0.97]
Rubing Pan 2020 A	4.13423941	0.311993	24	3.76963409	0.57218401	23	4.2%	0.78 [0.19, 1.38]
Rubing Pan 2020 B	4.0704356	0.405269	25	3.76963409	0.57218401	23	4.2%	0.60 [0.02, 1.18]
**Subtotal (95% CI)**			**333**			**270**	**39.3%**	**0.18 [-0.26, 0.61]**
Heterogeneity: Tau² = 0.36; Chi² = 49.26, df = 8 (P < 0.00001); I² = 84%								
Test for overall effect: Z = 0.81 (P = 0.42)								
**9.4.4 V3-V5**								
Sushrut Jangi 2016 A	4.57188911	0.349666	61	4.4120891	0.30890159	44	5.3%	0.48 [0.08, 0.87]
**Subtotal (95% CI)**			**61**			**44**	**5.3%**	**0.48 [0.08, 0.87]**
Heterogeneity: Not applicable								
Test for overall effect: Z = 2.37 (P = 0.02)								
**9.4.5 V4**								
Isabelle Mack 2016	3.64014386	0.579847	99	3.42274765	0.61106109	59	5.7%	0.37 [0.04, 0.69]
Jessica D Forbes 2018	4.05020292	0.472551	40	4.27249137	0.3398189	47	5.1%	-0.54 [-0.97, -0.11]
Siobhán Ní Choileáin 2019	3.41819868	0.460231	26	3.7435243	0.48204265	39	4.6%	-0.68 [-1.19, -0.17]
Sushrut Jangi 2016 B	3.62295946	0.459037	61	3.60443513	0.4542453	44	5.3%	0.04 [-0.35, 0.43]
Tanya T. Nguyen 2019	3.37860676	0.680134	73	3.58207035	0.52592878	73	5.7%	-0.33 [-0.66, -0.01]
Xia Cao 2021	3.89177459	0.212122	8	4.0207517	0.23722116	41	3.3%	-0.54 [-1.31, 0.22]
Zhou Dan 2020	1.31497006	0.311741	143	1.39398292	0.31825954	143	6.2%	-0.25 [-0.48, -0.02]
**Subtotal (95% CI)**			**450**			**446**	**36.0%**	**-0.23 [-0.50, 0.03]**
Heterogeneity: Tau² = 0.09; Chi² = 20.50, df = 6 (P = 0.002); I² = 71%								
Test for overall effect: Z = 1.72 (P = 0.09)								
**9.4.6 V5**								
Severin Weis 2019	4.46584016	0.427696	156	4.55164959	0.355219	100	6.1%	-0.21 [-0.47, 0.04]
**Subtotal (95% CI)**			**156**			**100**	**6.1%**	**-0.21 [-0.47, 0.04]**
Heterogeneity: Not applicable								
Test for overall effect: Z = 1.66 (P = 0.10)								
**Total (95% CI)**			**1081**			**936**	**100.0%**	**-0.03 [-0.22, 0.17]**
Heterogeneity: Tau² = 0.15; Chi² = 84.94, df = 20 (P < 0.00001); I² = 76%								
Test for overall effect: Z = 0.27 (P = 0.79)								
Test for subgroup differences: Chi² = 11.80, df = 5 (P = 0.04), I² = 57.6%								

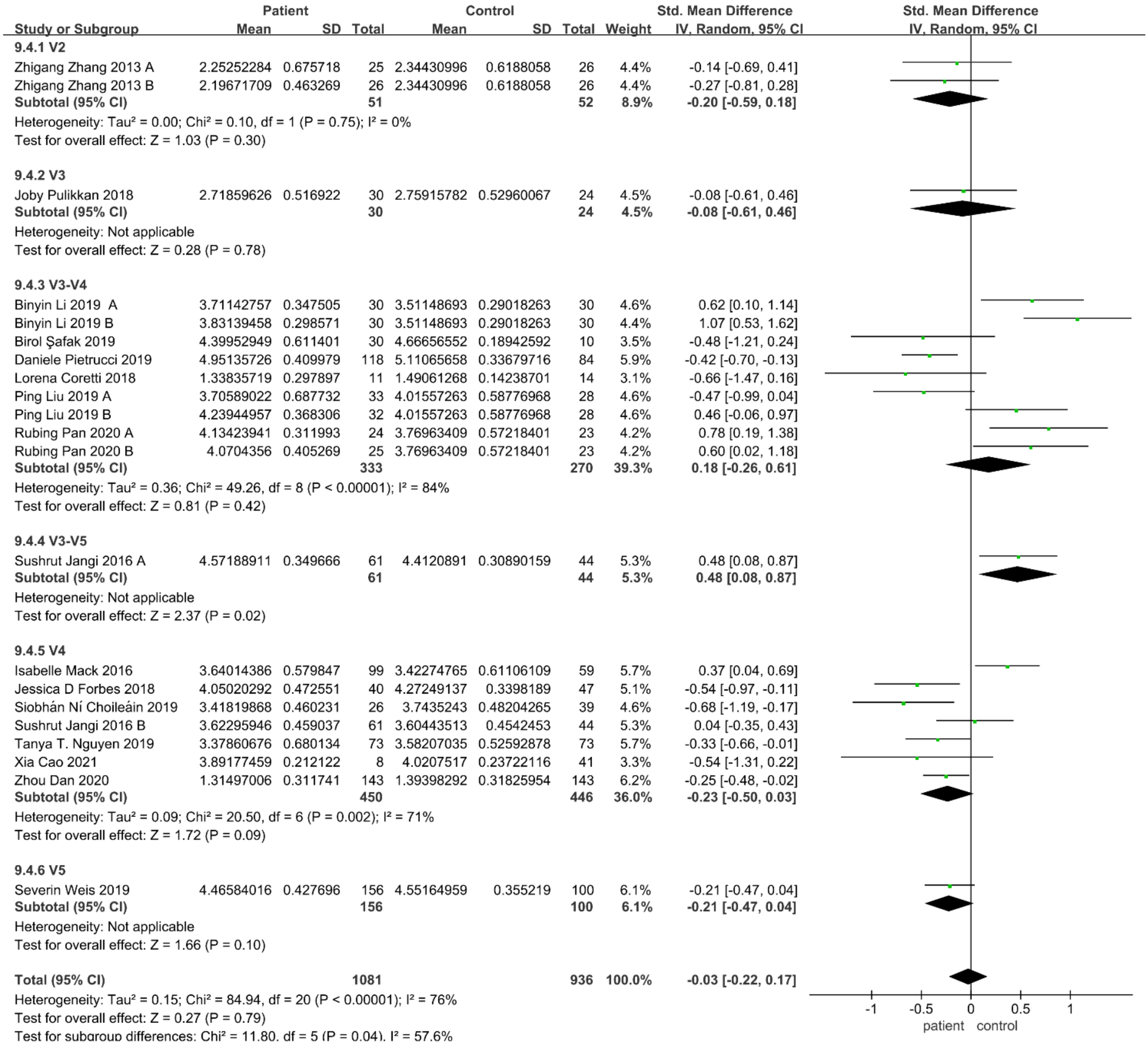

Supplementary Figure 5. Forest plot of the Shannon for the sequencing region. Abbreviations: SMD, standardized mean difference; SD, standard deviation.

Study or Subgroup	Patient Mean	Patient SD	Patient Total	Control Mean	Control SD	Control Total	Weight	Std. Mean Difference IV, Random, 95% CI
**9.5.1 V2**								
Zhigang Zhang 2013 A	0.802544	0.170379	25	0.840322	0.110268	26	4.3%	-0.26 [-0.81, 0.29]
Zhigang Zhang 2013 B	0.831332	0.117622	26	0.840322	0.110268	26	4.3%	-0.08 [-0.62, 0.47]
**Subtotal (95% CI)**			**51**			**52**	**8.6%**	**-0.17 [-0.55, 0.22]**
Heterogeneity: Tau² = 0.00; Chi² = 0.21, df = 1 (P = 0.64); I² = 0%								
Test for overall effect: Z = 0.85 (P = 0.40)								
**9.5.2 V3**								
Joby Pulikkan 2018	0.836505	0.1186	30	0.849823	0.082381	24	4.4%	-0.13 [-0.66, 0.41]
**Subtotal (95% CI)**			**30**			**24**	**4.4%**	**-0.13 [-0.66, 0.41]**
Heterogeneity: Not applicable								
Test for overall effect: Z = 0.46 (P = 0.65)								
**9.5.3 V3-V4**								
Binyin Li 2019 A	0.96742	0.014119	30	0.960637	0.011909	30	4.5%	0.51 [-0.00, 1.03]
Binyin Li 2019 B	0.972584	0.011957	30	0.960637	0.011909	30	4.4%	0.99 [0.45, 1.53]
Birol Şafak 2019	0.978754	0.02182	30	0.988481	0.002289	10	3.3%	-0.50 [-1.22, 0.23]
Daniele Pietrucci 2019	0.98766	0.006604	118	0.98972	0.005154	84	6.2%	-0.34 [-0.62, -0.06]
Lorena Coretti 2018	0.712727	0.104028	11	0.765714	0.041084	14	2.9%	-0.68 [-1.50, 0.13]
Ping Liu 2019 A	0.954275	0.038126	33	0.968519	0.027939	28	4.6%	-0.42 [-0.92, 0.09]
Ping Liu 2019 B	0.978002	0.010758	32	0.968519	0.027939	28	4.5%	0.45 [-0.06, 0.97]
Rubing Pan 2020 A	0.977219	0.009476	24	0.964406	0.028404	23	4.1%	0.60 [0.01, 1.19]
Rubing Pan 2020 B	0.97458	0.014541	25	0.964406	0.028404	23	4.2%	0.45 [-0.12, 1.02]
**Subtotal (95% CI)**			**333**			**270**	**38.7%**	**0.14 [-0.25, 0.52]**
Heterogeneity: Tau² = 0.26; Chi² = 38.21, df = 8 (P < 0.00001); I² = 79%								
Test for overall effect: Z = 0.70 (P = 0.48)								
**9.5.4 V3-V5**								
Sushrut Jangi 2016 A	0.982433	0.009915	61	0.979635	0.008913	44	5.4%	0.29 [-0.10, 0.68]
**Subtotal (95% CI)**			**61**			**44**	**5.4%**	**0.29 [-0.10, 0.68]**
Heterogeneity: Not applicable								
Test for overall effect: Z = 1.47 (P = 0.14)								
**9.5.5 V4**								
Isabelle Mack 2016	0.924276	0.079449	99	0.890296	0.106682	59	5.9%	0.37 [0.05, 0.70]
Jessica D Forbes 2018	0.965733	0.023717	40	0.976369	0.008744	47	5.1%	-0.61 [-1.04, -0.18]
Siobhán Ní Choileáin 2019	0.92643	0.041201	26	0.944311	0.034393	39	4.6%	-0.47 [-0.98, 0.03]
Sushrut Jangi 2016 B	0.926337	0.054094	61	0.933155	0.052588	44	5.4%	-0.13 [-0.51, 0.26]
Tanya T. Nguyen 2019	0.895817	0.110787	73	0.921409	0.043338	73	5.9%	-0.30 [-0.63, 0.02]
Xia Cao 2021	0.970678	0.010062	8	0.976781	0.005662	41	3.0%	-0.92 [-1.70, -0.14]
Zhou Dan 2020	0.652753	0.116871	143	0.676355	0.111834	143	6.5%	-0.21 [-0.44, 0.03]
**Subtotal (95% CI)**			**450**			**446**	**36.5%**	**-0.26 [-0.52, 0.01]**
Heterogeneity: Tau² = 0.09; Chi² = 20.35, df = 6 (P = 0.002); I² = 71%								
Test for overall effect: Z = 1.90 (P = 0.06)								
**9.5.6 V5**								
Severin Weis 2019	0.980233	0.011797	156	0.982642	0.008301	100	6.4%	-0.23 [-0.48, 0.02]
**Subtotal (95% CI)**			**156**			**100**	**6.4%**	**-0.23 [-0.48, 0.02]**
Heterogeneity: Not applicable								
Test for overall effect: Z = 1.77 (P = 0.08)								
**Total (95% CI)**			**1081**			**936**	**100.0%**	**-0.06 [-0.24, 0.12]**
Heterogeneity: Tau² = 0.11; Chi² = 69.34, df = 20 (P < 0.00001); I² = 71%								
Test for overall effect: Z = 0.68 (P = 0.50)								
Test for subgroup differences: Chi² = 7.80, df = 5 (P = 0.17), I² = 35.9%								

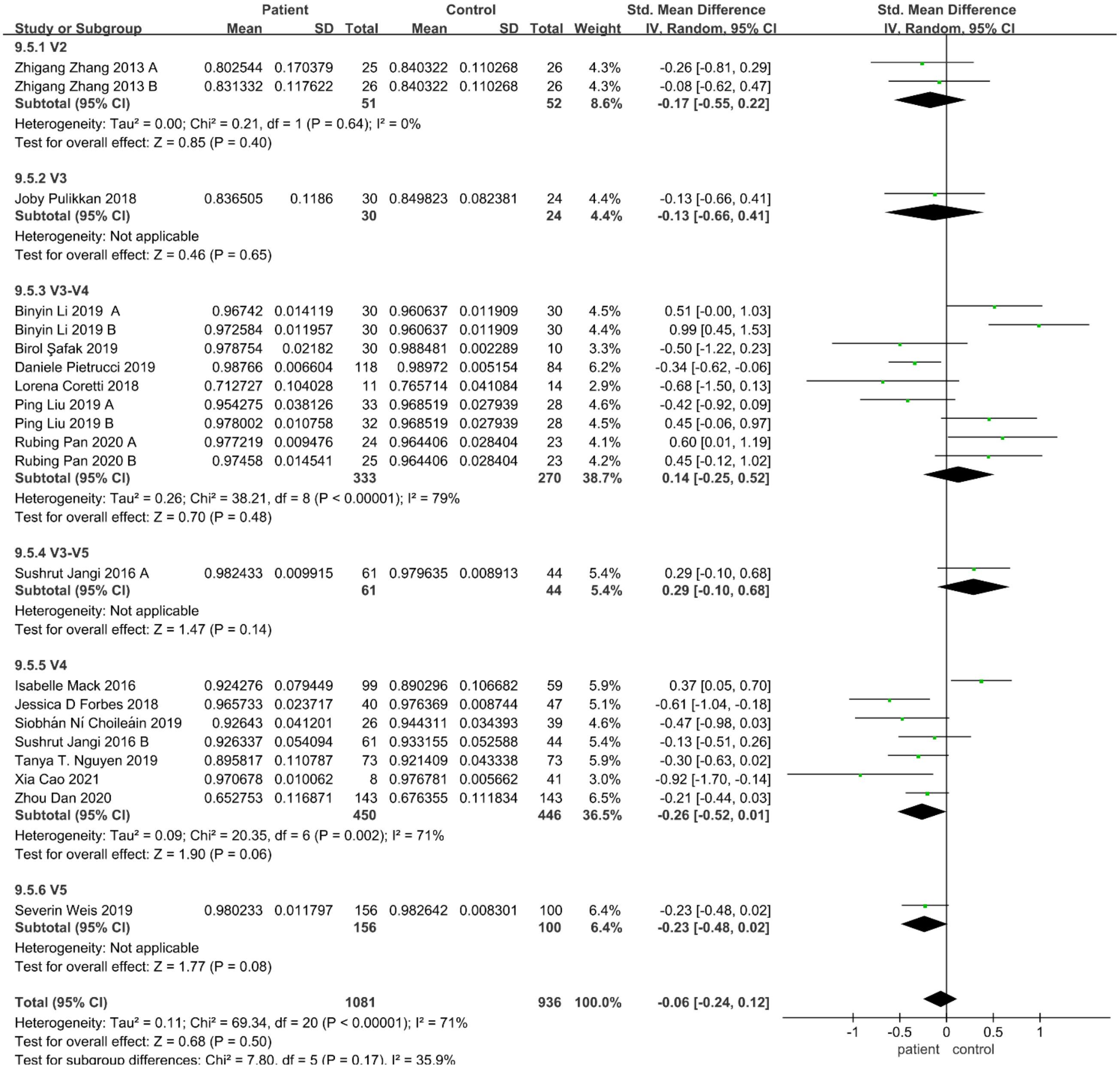

Supplementary Figure 6. Forest plot of the Simpson for the sequencing region. Abbreviations: SMD, standardized mean difference; SD, standard deviation.

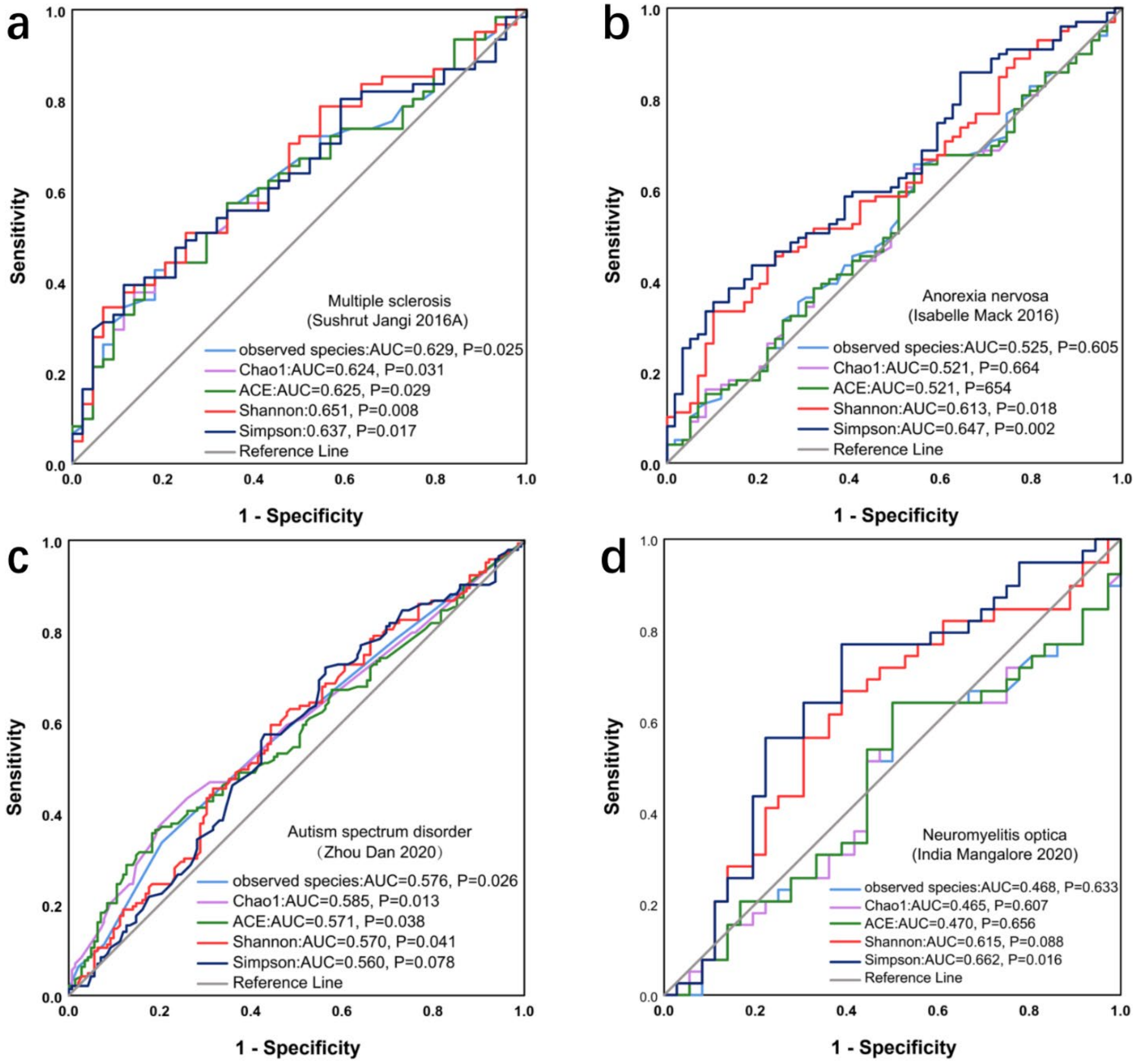

Supplementary Figure 7. ROC curve analysis on neurological diseases. ROC curve for Multiple sclerosis (a), Anorexia nervosa (b), Autism spectrum disorder (c) and Neuromyelitis optica (d). Abbreviations: ROC, receiver operating characteristic; AUC, area under the curve.
